# Synthesis and biological evaluation of stilbene derivatives coupled to NO donors as potential antidiabetic agents

**DOI:** 10.1080/14756366.2018.1425686

**Published:** 2018-01-29

**Authors:** Bing Wang, Teng Liu, Zhongyu Wu, Lei Zhang, Jie Sun, Xiaojing Wang

**Affiliations:** a School of Medicine and Life Sciences, University of Jinan-Shandong Academy of Medical Sciences, Jinan, China;; b Institute of Materia Medica, Shandong Academy of Medical Sciences, Jinan, China;; c Key Laboratory for Biotech-Drugs Ministry of Health, Jinan, China;; d Key Laboratory for Rare & Uncommon Diseases of Shandong Province, Jinan, China

**Keywords:** α-Glucosidase inhibitor, antidiabetic, AGEs formation inhibitor, stilbene, nitric oxide donor

## Abstract

The work is focused on the design of drugs that prevent and treat diabetes and its complications. A novel class of stilbene derivatives were prepared by coupling NO donors of alkyl nitrate and were fully characterised by NMR and other techniques. These compounds were tested *in vitro* activity, including α-glucosidase inhibitory activity, aldose reductase (AR) inhibitory activity and advanced glycation end products (AGEs) formation inhibitory activity. A class of modified compounds could play a significant effect for treatment of diabetic complications. Target compounds **3e** and **7c** offered a potential drug design concept for the development of therapeutic or preventive agents for diabetes and its complications.

## 1. Introduction

Diabetes mellitus (DM) is a chronic, incurable metabolic disorder defined by the dysregulation of glucose homeostasis manifesting as hyperglycaemia, abnormalities in lipid and protein metabolism, and the development of both acute and long-term complications in target organs, including retina, kidney, peripheral nerves, and the cardiovascular system^1^. In 2014, the IDF estimated that 8.2% of adults aged 20–79 (387 million people) were living with diabetes, compares with 382 million people in 2013, and this number was projected to rise beyond 592 million in 2035^2^. Chronic hyperglycaemia is the primer of a series of cascade reactions causing the over production of free radicals and increasing evidences indicate that these contribute to the development of diabetic complications such as blindness, cardiac and kidney disease. Effective glycaemic control can lower the incidence of diabetic complications and reduce their severity.

Stilbene compounds such as rosewood and resveratrol have been shown to be effective in the treatment of diabetes. *Pterocarpus marsupium* Roxb. commonly known as vengisa or bijasal, is well known for its medicinal properties in Ayurvedic and Unani systems in the treatment of diabetes. The water extract of the heartwood and root shows good curative properties for diabetes, and this may be due to the presence of pterostilbene^3^^,^^4^. Pari and Satheesh^5^ reported that oral administration of pterostilbene decreased glucose levels in streptozotocin (STZ)-diabetic rats through long-term trials conducted in 2006. Resveratrol also shows a good candidate as a neutraceutical support for the therapy of obesity and type II diabetes^6^.

Nitric oxide (NO) is a short-lived free radical whose half-life is only a few seconds. It can easily pass through the cell membrane because of its small molecule and lipophilic properties^7^. Nitric oxide is synthesised from the amino acid l-arginine in a reaction mediated by one of several isoforms of the enzyme nitric oxide synthase (NOS)^8^, and it plays a very important role in mammalian physiology and pathophysiology^9^. The lack of NO relates to many pathologic processes, thus providing a solid biological basis for the use of NO replacement therapy. Exogenous NO sources constitute a powerful way to supply NO when the body fails to generate sufficient NO for normal biological functions. This theory opens up the possibility of designing new drugs that can deliver NO into tissues and the bloodstream in a sustained and controlled manner. This objective has been achieved by grafting an organic nitrate structure onto existing drugs through chemical spacers, such as aliphatic, aromatic, or a heterocyclic chain. The approach has led to the synthesis of several new chemical entities whose pharmacologic profile challenges the parent drug, not only on the basis of new properties, but also raised safety^10^. Great advances in knowledge on biochemical pathways and about pharmacological properties involving NO have sparked interest in devising hybrid molecules containing NO-donors that allow synergism of action between NO and the broad chemical diversity of “native” drugs^11^.

## 2. Results and discussion

### 2.1. Chemistry

**Scheme 1.**
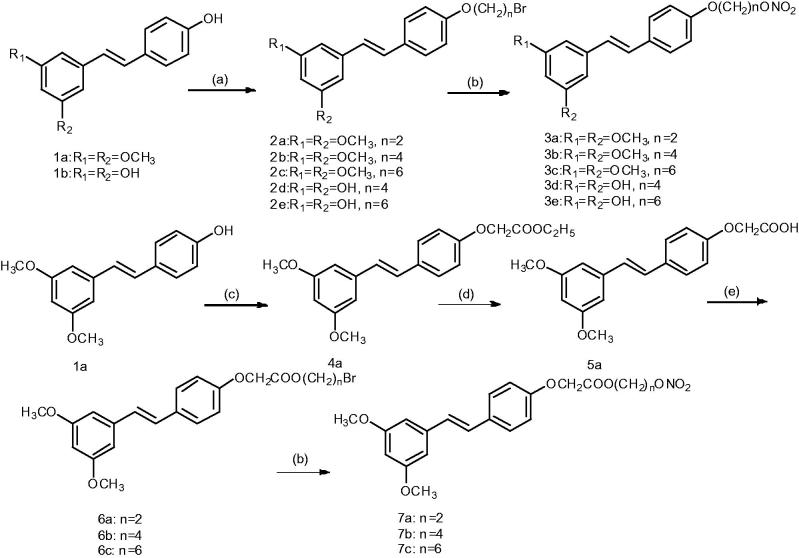
General synthetic route to NO-donor compounds **3a**–**3e** and **7a**–**7c**. Reagents and conditions: (a) dibromo alkane, K_2_CO_3_, acetone, reflux, 6 h; (b) AgNO_3_, acetonitrile, 80 °C, 2 h; (c) ethyl bromoacetate, K_2_CO_3_, acetone; (d) KOH, methanol; (e) dibromo alkane, Et_3_N, acetone, reflux, 24 h.

The preparation of the nitrate derivatives following the synthetic routes is summarised in Scheme 1. An alkyl chain with a bromide group was introduced to the 4′-position of stilbene **1a**–**b** by a selective reaction with an appropriate dibromo alkane, yielding the 4′-bromo alkane derivatives **2a**–**e**, which were subsequently converted to the nitro esters **3a**–**e** via treatment with AgNO_3_ (silver nitrate) in anhydrous acetonitrile. Details on the chemical and spectroscopic characterizations of compounds **3a**–**e** were described in the Supporting Information.

Stilbene **1a** was reacted with bromoacetate to produce compound **4a**. Subsequent hydrolysis of this compound and reaction with a dibromo alkane produced compounds **6a**–**c**. These compounds were then reacted with AgNO_3_, producing products **7a**–**c**. Details on the chemical and spectroscopic characterizations of compounds **7a**–**c** were described in the Supporting Information.

### 2.2. Biological evaluation

#### 2.2.1. *In vitro* NO releasing property^12^

As shown in Table 1, NO appeared to be released from all the stilbene derivatives upon incubation with phosphate-buffered saline solution (PBS at pH 7.4) in the presence of l-cysteine, indicating that all the stilbene derivatives were exogenous NO donor compounds. The percentage of released NO varied from 0.896 ± 0.004% to 1.391 ± 0.050%, indicating a slow release. The amount of NO released from sodium nitroprusside (SNP), however, was substantially higher (6.825 ± 0.187%). At higher concentrations, particularly under conditions of oxidative stress, NO is highly cytotoxic, a feature that is exploited by inflammatory cells in response to invading pathogens^13^. Some researchers demonstrated that NO could stimulate glucose metabolism or transport in adipocytes or skeletal muscle^14–16^. Those stilbene derivatives could release NO in a slow rate. We think this can be useful to treat diabetes. Further *in vivo* experiments showed that the compounds had no significant effect on blood pressure in normal mice (data not shown).

**Table 1.** Biological evaluation *in vitro*.

		IC_50_ value (μM)		
Products	NO releasing activity (%)^a^	α-Glucosidase inhibitory activity^b^	AGEs inhibitory activity^c^	ALR2 inhibitory activity^d^
DMSO				
Pterostilbene		>1000	1.99 ± 0.51**	>1000
Resveratrol		241.53 ± 16.98**	0.83 ± 0.21**	17.02 ± 0.31
**3a**	1.029 ± 0.009**	>1000	2.90 ± 0.09**	>1000
**3b**	1.359 ± 0.034**	>1000	2.39 ± 0.11**	>1000
**3c**	1.391 ± 0.050**	83.12 ± 16.21**	1.49 ± 0.20**	299.25 ± 22.62**
**3d**	0.896 ± 0.004**	>1000	1.39 ± 0.16**	10.03 ± 0.15
**3e**	0.900 ± 0.006**	178.74 ± 5.76**	1.05 ± 0.25**	8.18 ± 0.33
**7a**	0.922 ± 0.020**	>1000	1.29 ± 0.04**	>1000
**7b**	1.000 ± 0.004**	>1000	0.97 ± 0.16**	>1000
**7c**	1.056 ± 0.023**	163.40 ± 14.20**	0.68 ± 0.07**	121.13 ± 13.28**
SNP	6.825 ± 0.187			
Acarbose		0.05 ± 0.003		
AG			282.81 ± 8.52	
Quercetin				3.71 ± 0.29

Each value represents the mean ± SD (*n* = 3).

a Percentage of NO released relative to a theoretical maximum release of 1 mol NO/mol of test compound; determined by Griess reagent in the presence of 5 mM l-cysteine, at pH 7.4.

b The concentration required for a 50% inhibition in the optical density of PNP (p-nitrophenol) at 405 nm relative to DMSO. IC_50_ values were calculated from the dose inhibition curve.

c The concentration required for a 50% inhibition of the fluorescence intensity of AGE relative to 0.1% DMSO, IC_50_ values were calculated from the dose inhibition curve.

d The concentration required for a 50% inhibition of the decrease in the optical density of NADPH at 340 nm relative to DMSO. IC_50_ values were calculated from the dose inhibition curve.

** *p* < .01 significantly different ANOVA followed by Dunnett’s *t*-test for comparison with standard.

#### 2.2.2. *In vitro* α-glucosidase inhibitory activity^17^

α-Glucosidases, enzymes anchored in the brush border of the small intestine, are responsible for catalysing the hydrolysis of carbohydrates. Their inhibitors were useful for the treatment of type II DM^18^. As shown in Table 1, just three compounds (**3c**, **3e**, and **7c**) presented moderate inhibitory activity for α-glucosidase. Notably, **3c** (IC_50_ = 83.12 ± 16.21 µM) had relatively strong activity which displayed weaker capacity than Acarbose (IC_50_=0.05 ± 0.003 µM). None of the linker with 1,2-dibromoethane or 1,4-dibromobutane showed good inhibitory activity, indicating that increasing the length of linker in NO donor compounds was necessary for inhibiting α-glucosidase. Through comparison of the IC_50_ values, **7c**>**3c**, showed that linker of NO donor compounds with carbonyl group reduced α-glucosidase inhibitory activity. Through comparison of the IC_50_ values, **3d**>**3e**, showed that the length of linker in NO donor compounds played an important role in enhancing the α-glucosidase inhibitory activity.

#### 2.2.3. *In vitro* inhibitory activity of AGEs (advanced glycation end products) formation^19^^,^^20^

Advanced glycation end products are a group of complex and heterogeneous compounds, which are implicated in a number of biochemical abnormalities associated with diabetes^21^^,^^22^. Therefore, the discovery of AGEs inhibitors would be beneficial to the prevention and treatment of diabetic or other pathogenic complications^23^. The results listed in Table 1 displayed that most of the target compounds presented strong inhibitory activity to AGEs, even better than amino guanidine (AG) (IC_50_ = 282.81 ± 8.52 µM). Through comparison of the IC_50_ values, **7c**<**7 b**<**3e**<**7a**<**3d**<**3c**<1.5 µM, those compounds were over 100 folds compared with positive reference AG in inhibitory activity to the formation of AGEs. The AGEs inhibitory capacities of **3d**>**3b**, **3e**>**3c**, indicate that absence of 3,5-dihydroxy groups in stilbene compounds enhanced their inhibitory activity to the formation of AGEs. The AGEs inhibitory capacities of **7b**>**3b**, **7c**>**3c**, indicate that linker with carbonyl group in NO donor compounds enhanced their inhibitory activity to the formation of AGEs.

#### 2.2.4. *In vitro* ALR2 inhibitory activity^24^

The enzyme aldose reductase (ALR2) is a member of the aldo–keto reductase superfamily. The development and progression of chronic diabetic complications are confirmed to be quite related to the activation and/or over expression of ALR2^25^. Therefore, ALR2 inhibitors may play a critical role in preventing or treating these complications. The results listed in Table 1 displayed that the target compounds **3c**, **3d**, **3e**, and **7c** resulted in high inhibitory activities on ALR2 (IC_50_ = 8.18–299.25 µM). The target compound **3d** and **3e** presented strong inhibitory activity to ALR2, showing little weaker inhibitory activity than quercetin (IC_50_ = 3.71 ± 0.29 µM). Target compounds **3d** and **3e** exerted higher ALR2 inhibition activities than the other serial compounds, indicating that absence of 3,5-dihydroxy groups in stilbene compounds was more effective in inhibiting ALR2. Target compounds **3c** and **7c** exerted weaker ALR2 inhibition activities, indicating that increasing the length of linker in NO donor compounds showed weak effect on enhancing their inhibitory activity to ALR2.

#### 2.2.5. Oral toxicity to mice

With reference to Lorke’s method^26^, Kunming mice were used as targets to estimate oral toxicity of each compound to mice. We select compounds **3c**, **3e**, and **7c** at concentrations of 10, 100, and 1000 mg/kg to test their oral toxicity in the first phase, and 1600, 2900, and 5000 mg/kg at the second phase. Results showed that none of the tested compounds significantly affected mice’ viability. No death and no appetite-suppressant effect were detected in the tested mice in 14 days. Since no death or damage was observed throughout the experiment, the LD_50_ was higher than 5000 mg/kg for the three compounds assayed, indicating their innocuousness for mice.

#### 2.2.6. Acute hypoglycaemic assay

**Figure 1.**
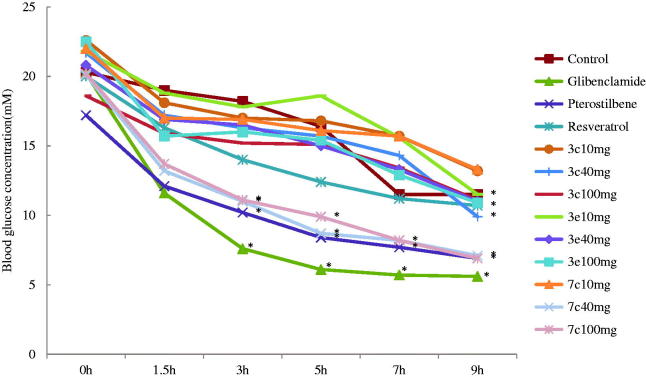
Acute effect of compounds **3c**, **3e**, and **7c** on blood glucose levels in STZ-diabetics mice. Each value is the mean ± SEM for six mice in each group. *p < .05 significantly different ANOVA followed by Dunnett’s *t*-test for comparison with respect to initial levels in each group.

The antidiabetic activity was determined by using a standard method^27^. As shown in Table 2, the target compounds **3c**, **3e**, and **7c** (40 mg/kg of bw) caused decreases in blood glucose levels in STZ-diabetic mice compared with normal mice (Table 2). Especially, compound **7c** caused significant decreases in blood glucose levels when compared with vehicle-treated groups (*p* < .05). The target compounds **3c** and **3e** showed similar antidiabetic activity throughout the experiment, their hypoglycaemic effect was weaker than pterostilbene at dose of 40 mg/kg of body weight. In STZ-diabetic animals, the hypoglycaemic effect of pterostilbene (40 mg/kg of bw) was larger than 50% since 5 h and persisted throughout the experiment. Target compound **7c** (40 mg/kg of bw) caused significant decreases in blood glucose levels compared with pterostilbene (40 mg/kg of bw). This result indicated that the NO donor was an adjunct to the hypoglycaemic effect in STZ-diabetic animal. However, glibenclamide (10 mg/kg of bw) which was used as a positive control, showed the strongest hypoglycaemic effect in STZ-induced diabetic mice than all compounds tested (Table 2). Target compound **7c** exerted higher hypoglycaemic effect than other serial compounds, indicating that linker with carbonyl group in NO donor compounds and the absence of 3,5-dimethoxy groups in stilbene compounds enhanced their hypoglycaemic effect (Figure 1).

**Table 2.** Acute effect of compounds **3c**, **3e**, and **7c** on blood glucose levels in STZ-diabetics mice.

Test samples	Blood glucose concentration (mM)						
	Dose (per os) mg/kg of bw	0 h	1.5 h	3 h	5 h	7 h	9 h
Control (vehicle)	–	20.3 ± 10.1	19.0 ± 5.6 (–6.16)	18.2 ± 5.2 (–10.36)	16.4 ± 6.4 (–19.00)	11.5 ± 5.5 (–43.17)	11.5 ± 6.4 (–43.17)
Glibenclamide	10	20.2 ± 5.4	11.6 ± 4.9 (–42.63)	7.6 ± 3.9* (–62.32)	6.1 ± 3.6* (–69.58)	5.7 ± 3.6* (–71.84)	5.6 ± 3.6* (–72.11)
Pterostilbene	40	17.2 ± 7.0	12.1 ± 6.4 (–29.91)	10.2 ± 6.2* (–40.89)	8.4 ± 5.6* (–51.42)	7.7 ± 5.3* (–55.36)	6.9 ± 4.3* (–59.82)
Resveratrol	40	20.0 ± 2.6	16.3 ± 2.9 (–18.42)	14.0 ± 2.0 (–29.83)	12.4 ± 2.1 (–38.08)	11.2 ± 1.8 (–44.25)	10.7 ± 1.4* (–46.33)
**3c**	10	22.6 ± 7.0	18.1 ± 7.3 (–20.13)	17.0 ± 6.6 (–24.78)	16.8 ± 6.1 (–25.66)	15.7 ± 4.3 (–30.53)	13.2 ± 3.5 (–41.59)
**3c**	40	21.7 ± 5.7	17.2 ± 5.6 (–20.74)	16.3 ± 7.5 (–25.04)	15.7 ± 6.4 (–27.57)	14.3 ± 9.6 (–34.02)	9.9 ± 4.6* (–54.30)
**3c**	100	18.6 ± 1.9	15.9 ± 3.6 (–14.34)	15.2 ± 3.0 (–18.28)	15.1 ± 3.7 (–19.00)	13.4 ± 4.7 (–28.23)	11.1 ± 3.0 (–40.32)
**3e**	10	21.8 ± 6.3	18.8 ± 5.2 (–13.84)	17.8 ± 4.8 (–18.35)	18.6 ± 7.3 (–14.53)	15.6 ± 6.0 (–28.59)	11.5 ± 4.4* (–47.48)
**3e**	40	20.8 ± 7.6	16.9 ± 5.6 (–18.67)	16.5 ± 9.1 (–20.83)	15.0 ± 8.7 (–28.04)	13.3 ± 9.4 (–36.22)	11.0 ± 8.0 (–47.20)
**3e**	100	22.5 ± 5.6	15.7 ± 7.7 (–30.37)	16.0 ± 6.8 (–29.04)	15.4 ± 9.6 (–31.78)	12.9 ± 6.4 (–42.81)	10.9 ± 4.5 (–51.48)
**7c**	10	22.0 ± 5.2	17.0 ± 7.4 (–22.65)	16.9 ± 10.6 (–23.18)	16.1 ± 7.7 (–26.82)	15.7 ± 8.3 (–28.56)	13.3 ± 5.7 (–39.70)
**7c**	40	20.1 ± 1.9	13.2 ± 2.8 (–34.33)	11.0 ± 3.6* (–45.11)	8.7 ± 2.5* (–56.80)	8.2 ± 1.8* (–59.37)	7.1 ± 1.2* (–64.76)
**7c**	100	20.2 ± 2.0	13.7 ± 4.0 (–32.43)	11.1 ± 3.0* (–44.88)	9.9 ± 2.5* (–50.83)	8.2 ± 1.9* (–59.49)	6.9 ± 1.4* (–66.01)

Each value is the mean ± SEM for six mice in each group.

* *p* < .05 significantly different ANOVA followed by Dunnett’s *t*-test for comparison with respect to initial levels in each group.

% Variation of glycaemia is in parentheses.

## 3. Conclusions

A novel class of stilbene derivatives was prepared by coupling NO donors of alkyl nitrate. We then studied the pharmacological activity of the synthesised compounds. In the respect to its NO releasing property, all stilbene derivatives could release NO in a slow rate. In the respect to anti-diabetic complications, carbonyl group in the linker and the length of linker showed significant effect on treatment of diabetic complications. Notably, target compound **3e** had strong activity in inhibitory activity of AGEs formation, which displayed stronger capacity than AG. Compounds **3c**, **3e**, and **7c** showed relatively strong α-glucosidase inhibitory activity, which displayed weaker capacity than acarbose. Oral toxicity tests indicated their innocuousness for mice. We selected compounds **3c**, **3e**, and **7c** to investigate their antidiabetic activity *in vivo*. The results showed the effect of the target compound **7c** showed the strongest hypoglycaemic effect in STZ-induced diabetic mice which was weaker than that of the glibenclamide used as a positive control *in vivo*. The result of antidiabetic activity *in vivo* indicated that linker with carbonyl group in NO donor compounds enhanced their hypoglycaemic effect. In conclusion, the target compounds **3e** and **7c** offered a potential drug design concept for the development of therapeutic or preventive agents for diabetes and complications of diabetes.

## 4. Experimental

### 4.1. Synthesis

#### 4.1.1. Materials and methods

Melting points were determined using a Thiele tube and were uncorrected. The FT-IR spectra were recorded using a Thermo-Nicolet Nexus 670 spectrometer with KBr pellets. The ^1^H NMR and ^13^C NMR spectra were recorded with a Bruker AM-600 spectrometer (Billerica, MA) with TMS as the internal standard. Chemical shifts were reported at room temperature on a scale (ppm) with DMSO-d_6_ as the solvents and *J* values are given in hertz.

Mass spectra were obtained with an Agilent Trap VL LC/MS spectrometer (Santa Clara, CA). The absorbance was recorded by a Hitachi U-3000 UV spectrophotometer (Tokyo, Japan). Column chromatography was performed on silica gel (200–300 mesh). Unless otherwise noted, all solvents and reagents were commercially available and used without further purification.

#### 4.1.2. General method for synthesis of compounds 2a–e

Anhydrous potassium carbonate (0.69 g, 5.0 mmol) was added to a solution of **1a** (2.56 g, 10 mmol) in 50 ml of anhydrous acetone, followed by refluxing until the solution became clear. Then, 1,2-dibromo ethylene (9.40 g, 50 mmol) was added dropwise, followed by refluxing for 24 h and vacuum filtration. This procedure yielded concentrated filter liquor, from which a white solid was obtained. The solids were collected, washed with petroleum, 1% NaOH and water, respectively, then dried. Compounds **2d**–**2e** were obtained using the same procedures.

#### 4.1.3. General method for synthesis of compounds 3a–e

Compound **2a** (3.63 g, 10 mmol) was dissolved in 50 ml of anhydrous acetonitrile, followed by heating to 50 °C. AgNO_3_ (1.70 g, 10 mmol) and acetonitrile (20 ml) were added. The mixture was stirred by heating to 80 °C in the dark for 2 h. The precipitate was filtered out, and filtrate was concentrated. When cooling at room temperature, white crystals were precipitated. The filtrate was vacuum filtered and the resulting solid was washed three times with water then dried to obtain white crystal **3a**. Compounds **3b**–**3e** were obtained using the same procedures.

##### 4.1.3.1. O4′-Nitrooxyethyl pterostilbene (3a)

 White solid, yield: 60%, mp 142.2–143.2 °C, IR (KBr, *ν*, cm^−1^): 2966 (–CH_3_); 1624 (–ONO_2_); 1592, 1513, 1455, 1040, 960, 829 (Ar); 1238, 1149 (C–O). ^1^H NMR (600 MHz, DMSO-d_6_) *δ* (ppm): 3.78 (s, 6H), 4.34 (m, 2H), 4.89 (m, 2H), 6.40 (t, *J*  =  2.2 Hz, 1H), 6.75 (d, *J*  =  2.2 Hz, 2H), 6.98 (d, *J*  =  8.7 Hz, 2H), 7.05 (d, *J*  =  16.4 Hz, 1H), 7.22 (d, *J*  =  16.4 Hz, 1H), 7.55 (d, *J*  =  8.7 Hz, 2H). ^13^C NMR (150 MHz, DMSO-d_6_) *δ* (ppm): 55.65, 64.51, 72.50, 100.01, 104.70, 115.25, 127.00, 128.36, 128.89, 130.62, 139.84, 158.04 and 161.12. MS: *m*/*z* (%): 346.0 [M + 1]^+^, 269.8, 240.8.

##### 4.1.3.2. O4′-Nitrooxyethyl pterostilbene (3b)

White solid, yield: 70%, mp 110.9–111.3 °C, IR (KBr, *ν*, cm^−1^): 2972 (–CH_3_); 1620 (–ONO_2_); 1593, 1514, 1455, 1024, 965, 832 (Ar); 1257, 1148 (C–O). ^1^H NMR (600 MHz, DMSO-d_6_) *δ* (ppm): 1.82 (m, 4H), 3.77 (s, 6H), 4.03 (t, *J*  =  5.9 Hz, 2H), 4.60 (t, *J*  =  6.2 Hz, 2H), 6.39 (t, *J*  =  2.2 Hz, 1H), 6.74 (d, *J*  =  2.2 Hz, 2H), 6.94 (d, *J*  =  8.7 Hz, 2H), 7.02 (d, *J*  =  16.4 Hz, 1H), 7.21 (d, *J*  =  16.4 Hz, 1H), 7.52 (d, *J*  =  8.7 Hz, 2H). ^13^C NMR (150 MHz, DMSO-d_6_) *δ* (ppm): 23.47, 25.42, 55.65, 67.33, 74.01, 99.95, 104.64, 115.17, 126.63, 128.31, 129.03, 130.04, 139.90, 158.77 and 161.12. MS: *m*/*z* (%):374.1 [M + 1]^+^, 298.0, 268.8, 253.9.

##### 4.1.3.3. O4′-Nitrooxyethyl pterostilbene (3c)

White solid, yield: 71%, mp 110.9–111.1 °C, IR (KBr, *ν*, cm^−1^): 2943 (–CH_3_); 1617 (–ONO_2_); 1592, 1513, 1458, 1038, 971, 831 (Ar); 1258, 1148 (C–O). ^1^H NMR (600 MHz, DMSO-d_6_) *δ* (ppm): 1.43 (dd, *J*  =  15.2, 9.9 Hz, 4H), 1.70 (m, 4H), 3.77 (s, 6H), 3.98 (t, *J*  =  5.6 Hz, 2H), 4.53 (t, *J*  =  6.1 Hz, 2H), 6.39 (s, 1H), 6.74 (s, 2H), 6.93 (d, *J*  =  8.0 Hz, 2H), 7.01 (d, *J*  =  16.4 Hz, 1H), 7.20 (d, *J*  =  16.3 Hz, 1H), 7.51 (d, *J*  =  7.9 Hz, 2H). ^13^C NMR (150 MHz, DMSO-d_6_) *δ* (ppm): 25.32, 25.57, 26.45, 28.94, 55.65, 67.81, 74.28, 99.93, 104.64, 115.14, 126.55, 128.30, 129.06, 129.90, 139.92, 158.93 and 161.11. MS: *m*/*z* (%):402.3 [M + 1]^+^, 356.0, 326.0, 268.9, 240.8, 204.8.

##### 4.1.3.4. O4′-Nitrooxyethyl resveratrol (3d)

White solid, yield: 46%, mp 140.4–141.3 °C, IR (KBr, *ν*, cm^−1^): 3386 (OH); 1625 (–ONO_2_); 1574, 1512, 1446, 963, 832 (Ar); 1238, 1146 (C–O). ^1^H NMR (600 MHz, DMSO-d_6_) *δ* (ppm): 1.43 (m, 4H), 1.70 (m, 4H), 3.97 (t, *J*  =  6.2 Hz, 2H), 4.52 (t, *J*  =  6.5 Hz, 2H), 6.14 (s, 1H), 6.41 (s, 2H), 6.93 (m, 4H), 7.49 (d, *J*  =  7.8 Hz, 2H), 9.21 (s, 2H). ^13^C NMR (150 MHz, DMSO-d_6_) *δ* (ppm): 25.32, 25.57, 26.44, 28.94, 31.15, 67.79, 74.27, 102.39, 104.86, 115.07, 127.05, 127.95, 128.22, 130.01, 139.56, 158.77 and 158.98. MS: *m*/*z* (%):346.0 [M + 1]^+^, 282.0, 240.8.

##### 4.1.3.5. O4′-Nitrooxyethyl resveratrol (3e)

White solid, yield: 69%, mp 134.2–135.9 °C, IR (KBr, *ν*, cm^−1^): 3491 (OH); 1606 (–ONO_2_); 1573, 1512, 964, 834 (Ar); 1255, 1149 (C–O). ^1^H NMR (600 MHz, DMSO-d_6_) *δ* (ppm): 1.43 (m, 4H), 1.70 (m, 4H), 3.97 (t, *J*  =  6.5 Hz, 2H), 4.52 (t, *J*  =  6.6 Hz, 2H), 6.13 (t, *J*  =  2.1 Hz, 1H), 6.40 (d, *J*  =  2.1 Hz, 2H), 6.92 (m, 4H), 7.49 (d, *J*  =  8.8 Hz, 2H), 9.20 (s, 2H). ^13^C NMR (150 MHz, DMSO-d_6_) *δ* (ppm): 25.33, 25.57, 26.45, 28.94, 67.79, 74.28, 102.38, 104.86, 115.07, 127.05, 127.95, 128.23, 130.01, 139.56, 158.77 and 158.98. MS: *m*/*z* (%):374.2 [M + 1]^+^, 328.0, 204.8.

#### 4.1.4. General method for synthesis of compounds 6a–c

A mixture of 4-methoxybenzaldehyde **1a** (200 mmol), malonic acid (240 mmol) and piperidine (2 ml) in pyridine (50 ml) was heated to reflux for 8 h at 90 °C. After the reaction is completed, hydrochloric acid solution (150 ml, 3 mol/l) was added. Then filtered to obtain a white crude product 24 h later. The crude product was recrystallised from absolute ethanol to afford 4-methoxyphenylacrylic acid **2a**.

Anhydrous potassium carbonate (0.69 g, 5.0 mmol) was added to a solution of **1a** (2.56 g, 10 mmol) in 50 ml of anhydrous acetone, followed by refluxing until the solution became clear. Then, ethyl bromoacetate (2.3 ml, 20 mmol) was added dropwise, followed by refluxing for 24 h and vacuum filtration. This procedure yielded concentrated filter liquor, from which a white solid was obtained. The solids were collected, washed with petroleum, 1% NaOH and water, respectively, then dried to obtain white solid **4a** (3.16 g, in 92% yield).

Potassium hydroxide (3.36 g, 60 mmol) was added to a solution of **4a** (3.42 g, 10 mmol) in anhydrous methanol (150 ml). The solution was then mechanically stirred and heat refluxed for 3 h until a white solid that does not dissolve in methanol was obtained. The solid dissolved in water. Then, the pH was adjusted to the desired acidity with hydrochloric acid, a white solid that does not dissolve in water was obtained. The solution underwent vacuum filtration, washed with water, then dried to obtain white solid **5a** (2.78 g, in 89% yield).

Triethylamine (4.15 ml, 30 mmol) was added to a solution of **5a** (3.14 g, 10 mmol) in acetone (100 ml). The mixture was then refluxed for 30 min. 1,2-Dibromoethane (9.40 g, 50 mmol) was dribbled into the mixture, followed by refluxing for 8 h and filtration to remove precipitates, then concentrating *in vacuo* to obtain the crude product. The crude product was subjected to column chromatography (silica, EtOAc–PE, 1:4) to obtain purified compound **6a** (2.05 g, in 49% yield). Compounds **6b**–**6c** were obtained using the same procedures.

#### 4.1.5. General method for synthesis of compounds 7a–c

Compound **6a** (2.11 g, 5 mmol) was dissolved in 50 ml of anhydrous acetonitrile, followed by heating to 50 °C. AgNO_3_ (0.85 g, 5 mmol) and acetonitrile (20 ml) were added. The mixture was stirred by heating to 80 °C in the dark for 2 h. The precipitate was filtered out, and filtrate was concentrated. When cooling at room temperature, white crystals were precipitated. The filtrate was vacuum filtered and the resulting solid was washed three times with water then dried to obtain white solid **7a**. Compounds **7b**–**7c** were obtained using the same procedures.

##### 4.1.5.1. O4′-Nitrooxyethyl pterostilbene (7a)

White solid, yield: 51%, mp 95.2–96.3 °C, IR (KBr, *ν*, cm^−1^): 2941 (–CH_3_); 1743 (C=O); 1613 (–ONO_2_); 1591, 1510, 1456, 977, 836 (Ar); 1257, 1149 (C–O). ^1^H NMR (600 MHz, DMSO-d_6_) *δ* (ppm): 3.77 (s, 6H), 4.47 (m, 2H), 4.77 (m, 2H), 4.86 (s, 2H), 6.39 (t, *J*  =  2.1 Hz, 1H), 6.75 (d, *J*  =  2.1 Hz, 2H), 6.96 (d, *J*  =  8.7 Hz, 2H), 7.04 (d, *J*  =  16.4 Hz, 1H), 7.22 (d, *J*  =  16.4 Hz, 1H), 7.53 (d, *J*  =  8.7 Hz, 2H). ^13^C NMR (150 MHz, DMSO-d_6_) *δ* (ppm): 54.59, 60.01, 63.93, 70.86, 98.98, 103.63, 114.24, 126.01, 127.17, 127.77, 129.75, 138.75, 156.65, 160.05 and 168.01. MS: *m*/*z* (%):404.0 [M + 1]^+^, 327.9, 268.8, 253.8.

##### 4.1.5.2. O4′-Nitrooxyethyl pterostilbene (7b)

White solid, yield: 76%, mp 124.3–124.9 °C, IR (KBr, *ν*, cm^−1^): 2947 (–CH_3_); 1747 (C=O); 1628 (–ONO_2_); 1593, 1512, 1450, 975, 841 (Ar); 1239, 1148 (C–O). ^1^H NMR (600 MHz, DMSO-d_6_) *δ* (ppm): 1.69 (m, 4H), 3.77 (s, 6H), 4.16 (t, *J*  =  5.8 Hz, 2H), 4.52 (t, *J*  =  6.0 Hz, 2H), 4.83 (s, 2H), 6.39 (t, *J*  =  2.1 Hz, 1H), 6.74 (d, *J*  =  2.1 Hz, 2H), 6.95 (d, *J*  =  8.7 Hz, 2H), 7.04 (d, *J*  =  16.4 Hz, 1H), 7.21 (d, *J*  =  16.4 Hz, 1H), 7.53 (d, *J*  =  8.7 Hz, 2H). ^13^C NMR (150 MHz, DMSO-d_6_) *δ* (ppm): 23.17, 24.89, 31.16, 55.65, 64.41, 65.09, 73.77, 100.03, 104.69, 115.24, 127.05, 128.25, 128.84, 130.75, 139.83, 157.81, 161.12 and 169.23. MS: *m*/*z* (%): 432.5 [M + 1]^+^, 356.4, 241.3.

##### 4.1.5.3. O4′-Nitrooxyethyl pterostilbene (7c)

White solid, yield: 79%, mp 98.0–99.1 °C, IR (KBr, *ν*, cm^−1^): 2900 (–CH_2_); 1765 (C=O); 1639 (–ONO_2_); 1591, 1513, 1456, 973, 841 (Ar); 1205, 1155 (C–O). ^1^H NMR (600 MHz, DMSO-d_6_) *δ* (ppm): 1.31 (m, 4H), 1.60 (m, 4H), 3.77 (s, 6H), 4.12 (t, *J*  =  6.5 Hz, 2H), 4.48 (t, *J*  =  6.6 Hz, 2H), 4.82 (s, 2H), 6.39 (t, *J*  =  1.9 Hz, 1H), 6.74 (d, *J*  =  2.1 Hz, 2H), 6.94 (d, *J*  =  8.7 Hz, 2H), 7.04 (d, *J*  =  16.4 Hz, 1H), 7.21 (d, *J*  =  16.4 Hz, 1H), 7.53 (d, *J*  =  8.7 Hz, 2H). ^13^C NMR (150 MHz, DMSO-d_6_) *δ* (ppm): 25.13, 25.27, 26.38, 28.32, 55.65, 64.85, 65.11, 74.20, 100.04, 104.67, 115.23, 127.03, 128.24, 128.82, 130.73, 139.82, 157.82, 161.11 and 169.27. MS: *m*/*z* (%):460.8 [M + 1]^+^, 414.4, 384.5.

### 4.2. Biological activity

#### 4.2.1. Animals

Normoglycemic Kunming mice, weight 18–20 g, were obtained from Jinan PengYue Experimental Animal Co., Ltd. (License number: SCXK (Lu) 2014-0007). The animals were housed under standard laboratory conditions and maintained on a standard pellet diet and water *ad libitum*. All experiments involving living animals and their care were performed in strict accordance with the National Care and Use of Laboratory Animals by the National Animal Research Authority (China) and guidelines of Animal Care and Use issued by University of Jinan Institutional Animal Care and Use Committee. The experiments were approved by the Institutional Animal Care and Use Committee of the School of Medicine and Life Sciences, University of Jinan. All efforts were made to minimise animal’s suffering and to reduce the number of animals used.

#### 4.2.2. Detection of nitrite

A solution of the appropriate compound (80 µL) in dimethyl sulphoxide (DMSO) was added to 8 ml of 1:1 v/v mixture of 50 mM PBS (pH 7.4) with MeOH, containing 5 × 10^−4^ M l-cysteine. The final concentration of target compounds was 10^−4^ M. After 1 h at 37 °C in dark, the reaction mixture was treated with 2 ml of the Griess reagent [sulphanilamide (1 g), N-naphthylethylenediamine dihydrochloride (0.1 g), 85% phosphoric acid (2.5 ml) in distilled water (final volume: 100 ml)]. After 10 min at 37 °C in dark, the absorbance was measured at 540 nm. Sodium nitrite standard solutions (1–80 µmol/ml) were used to construct the calibration curve. The results were expressed as the percentage of NO released (*n*  =  3) relative to a theoretical maximum release of 1 mol NO/mol of test compound.

#### 4.2.3. Influence on blood pressure

Influence on blood pressure was analysed in adult normotensive mice (25–30 g). After one week of acclimation, mean arterial pressure values were measured using the tail-cuff method with a blood pressure monitor (BP-2010, Softron Beijing Biotechnology Co., Ltd., Beijing, China) from 0 h to 8 h after administration of the standard and test compounds^28^. The test compounds (40 mg/kg and100 mg/kg) and standard (losartan, 20 mg/kg) were administered.

#### 4.2.4. *In vitro* α-glucosidase inhibitory activity

α-Glucosidase (G0660-750UN, Sigma Aldrich, St. Louis, MO) and 4-nitrophenyl α-d-glucopyranoside (PNPG, Macklin) were dissolved in phosphate buffer (pH 6.8, 100 mM), and the test compounds were dissolved in DMSO solution. The experiment was divided into blank group, control group, sample blank group and sample group. The reagents were loaded in 96-well plates at the dose of the table (Table 3). The solution was bathed in 37 °C water for 10 min, after the end, enzyme solution was added. After reaction at 37 °C for 20 min, 70 µL Na_2_CO_3_ solution (0.2 mM) was added to stop the reaction. All experiments were run in triplicate. Acarbose (Sigma Aldrich, St. Louis, MO) was used as a standard inhibitor^29^. Since PNPG can produce glucose and p-nitrophenol (PNP) under the action of α-glucosidase, PNP has the greatest absorption at 405 nm. The absorbance was determined by the microplate reader, and the inhibition rate of α-glucosidase and the IC_50_ value of each sample were calculated according to the formula.

$$\text{Inhibition rate }\%=\left\{\left[\left(A_{\text{C}}-A_{\text{B}}\right)-\left(A_{\text{S}}-A_{\text{SB}}\right)\right]/\left(A_{\text{C}}-A_{\text{B}}\right)\right\}\times 100\%$$

where *A*_C_ is the absorbance of control group; *A*_B_ is the absorbance of blank group; *A*_S_ is the absorbance of sample group; *A*_SB_ is the absorbance of sample blank group.

**Table 3.** The amount and order of each reactant of α-glucosidase inhibition test.

Reagents	Volume (μL)			
	Blank group	Control group	Sample blank group	Sample group
PBS	20	10	20	10
Compounds/Inhibitors	0	0	10	10
PNPG	20	20	20	20
Water	10	10	0	0
Mix well and incubate at 37 °C for 10 minutes				
α-Glucosidase	0	10	0	10
Mix well and react at 37 °C for 20 minutes				
Na_2_CO_3_	70	70	70	70

#### 4.2.5. *In vitro* inhibitory activity of AGEs formation

To prepare the AGE reaction solution, 10 mg/ml of bovine serum albumin in 50 mM PBS (pH 7.4) was added to 0.2 M glucose, and 0.02% sodium azide was added to prevent bacterial growth. The reaction mixture (3 ml) was then mixed with various concentrations (0.5–1000 µg/ml) of the target compounds (1 ml) dissolved in DMSO. After incubating at 37 °C for 14 d, the fluorescence intensity of AGE was determined by a fluorospectrophotometer (PE, Cincinnati, OH) with excitation and emission wavelengths at 350 nm and 420 nm^30^, respectively. All experiments were run in triplicate. Aminoguanidine hydrochloride was used as a reference compound. The inhibition rate of AGEs formation and the IC_50_ value of each sample were calculated according to the formula.

$$\text{Inhibition rate }\%=\left\{\left[\left(A_{\text{C}}-A_{\text{B}}\right)-\left(A_{\text{S}}-A_{\text{SB}}\right)\right]/\left(A_{\text{C}}-A_{\text{B}}\right)\right\}\times 100\%$$

where *A*_C_ is the absorbance of control group (1.0 ml glucose +1.0 ml bovine serum albumin +1.0 ml sodium azide +1.0 ml DMSO); *A*_B_ is the absorbance of blank group (1.0 ml PBS +1.0 ml bovine serum albumin +1.0 ml sodium azide +1.0 ml DMSO); *A*_S_ is the absorbance of sample group (1.0 ml glucose +1.0 ml bovine serum albumin +1.0 ml sodium azide +1.0 ml target compound solution or aminoguanidine hydrochloride solution); *A*_SB_ is the absorbance of sample blank group (1.0 ml PBS +1.0 ml bovine serum albumin +1.0 ml sodium azide +1.0 ml target compound solution or aminoguanidine hydrochloride solution).

#### 4.2.6. *In vitro* ALR2 inhibitory activity

After homogenisation and centrifugation, the crude ALR2 from mice was obtained^31^. The inhibitory activity of the compounds on ALR2 was carried out using crude enzyme and different concentrations of the compounds (1–1000 µg/ml) in 200 mM PBS (pH 6.2) containing 0.10 mM NADPH. The reaction was initiated by addition of glyceraldehyde and the decrease in the optical density of NADPH at 340 nm was recorded for 3 min. All experiments were run in triplicate. IC_50_ of the compounds was calculated. The flavonoid quercetin was used as a reference in the ALR2 assay.

#### 4.2.7. Oral toxicity to mice

Experiments were performed on Kunming mice (male and female half, body weight range, 25–30 g). Mice were housed in a climate and light controlled room with a 12 h light/dark cycle. Twelve hours before experiments, food was withheld, but animals had free access to drinking water. The compounds were suspended in vehicle (Tween-80, 0.2% in saline). The concentrations were adjusted to orally administrate 0.2 ml/10 g of bw (body weight). Mice were treated in two phases. In the first, intragastric doses of 10, 100 and 1000 mg/kg of bw of compounds were administered. On the second, the doses were adjusted to 1600, 2900 and 5000 mg/kg of bw of compounds. In both phases, mice were observed daily in a period of 14 days for mortality, toxic effects and/or changes in behavioural pattern. At the end of the experiments, the mice were sacrificed in a CO_2_ chamber.

#### 4.2.8. Acute hypoglycaemic assay

Type II DM was induced in mice by a single intraperitoneal injection of freshly prepared STZ (Sigma Aldrich, St. Louis, MO) dissolved in 0.1 M citrate buffer, pH 4.5, in a volume of 120 mg/kg of bw. After seven days of STZ administration, blood glucose levels of each mouse were determined. Mice with blood glucose levels higher than 11 mM were considered diabetic and were included in the study^32^.

STZ-induced diabetic mice and normal mice were placed in single cages with wire-net floors and deprived of food for 12 h before experimentation but allowed free access to tap water throughout. The compounds (at the doses of 10, 40, and 100 mg/kg of bw) were suspended in 0.05% Tween-80 in saline solution. Glibenclamide (10 mg/kg of bw) was suspended in the same vehicle. The target compounds were freshly prepared immediately before experimentation and administered by the intragastrical route at the doses of 10 ml/kg of bw. Control mice received only the vehicle (0.05% Tween-80 in saline solution) by the same route. Blood glucose levels were measured at 0, 1.5, 3, 5, 7, and 9 h after drugs administration^33^.

#### 4.2.9. Statistical analysis

Data were shown as mean ± SD. Differences between individual groups were analysed by using ANOVA followed by Dunett’s test. A difference with a *p* value of  < .05 was considered to be significant.

## Supplementary Material

IENZ_1425686_Supplementary_Material.pdf
