# Supplementary material for: Synthesis and biological evaluation of stilbene derivatives coupled to NO donors as potential antidiabetic agents
Source: J Enzyme Inhib Med Chem. 2018 Jan 29;33(1):416–23. doi: 10.1080/14756366.2018.1425686 (PMC7011920; doi:10.1080/14756366.2018.1425686)
Supplement: IENZ_1425686_Supplementary_Material.pdf [file IENZ_A_1425686_SM1431.pdf]

Synthesis, and biological evaluation of stilbene derivatives coupled to NO donors as potential antidiabetic agents

Bing Wang,[1,2,3,4] Teng Liu,[1,2,3,4] Zhongyu Wu, [1,2,3,4] Lei Zhang, [1,2,3,4] Jie Sun, [1,2,3,4]* and Xiaojing Wang [1,2,3,4]*

[1] *School of Medicine and Life Sciences, University of Jinan-Shandong Academy of Medical Sciences, Jinan 250200, Shandong, China*
[2] *Institute of MateriaMedica, Shandong Academy of Medical Sciences, Jinan 250062, Shandong, China*
[3] *Key Laboratory for Biotech-Drugs Ministry of Health, Jinan 250062, Shandong, China*
[4] *Key Laboratory for Rare & Uncommon Diseases of Shandong Province, Jinan 250062, Shandong, China*

*Corresponding authors: E-Mail:

E-Mail: sunjie310@126.com (J. Sun)

E-Mail: xiaojing6@gmail.com (X. Wang)

Supporting Information

Contents

IR(KBr) of compounds 3a-7c 2
HPLC spectra of compounds 3a-7c 6
Mass spectrum of compounds 3a-7c 10
^{1}H NMR(600 MHz, DMSO-d$_6$) of compounds 3a-7c 15
^{13}C NMR(150 MHz, DMSO-d$_6$) of compounds 3a-7c 19

IR(KBr) of compounds 3a-7c

3a

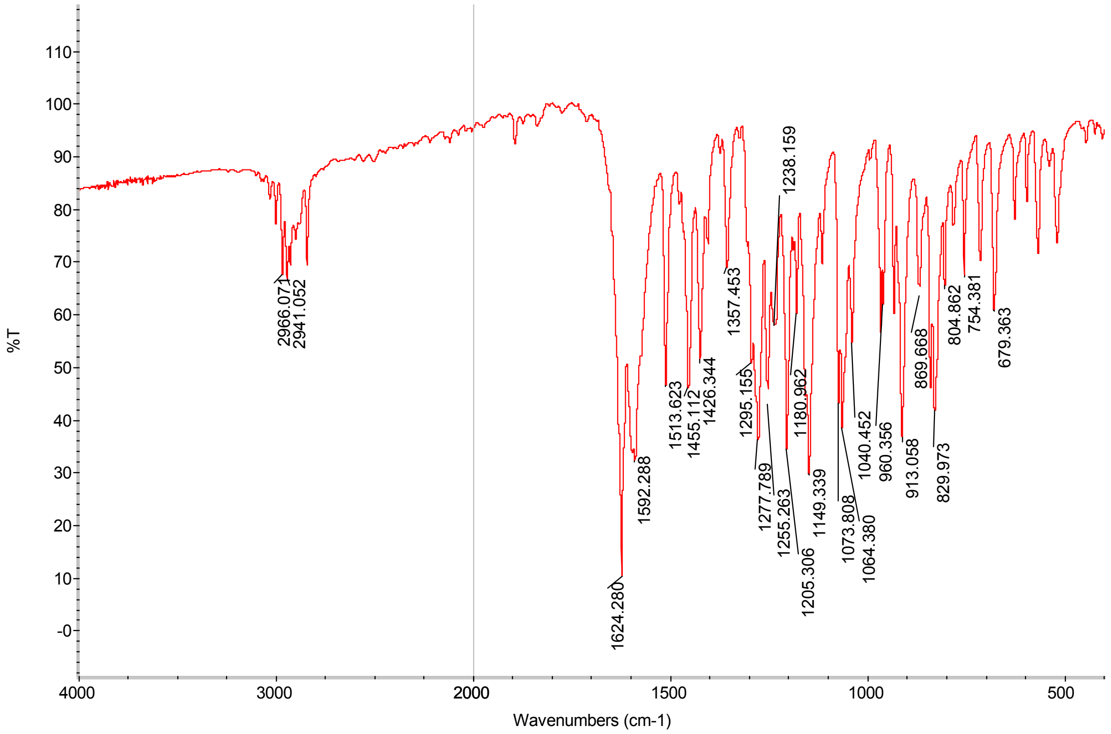

3b

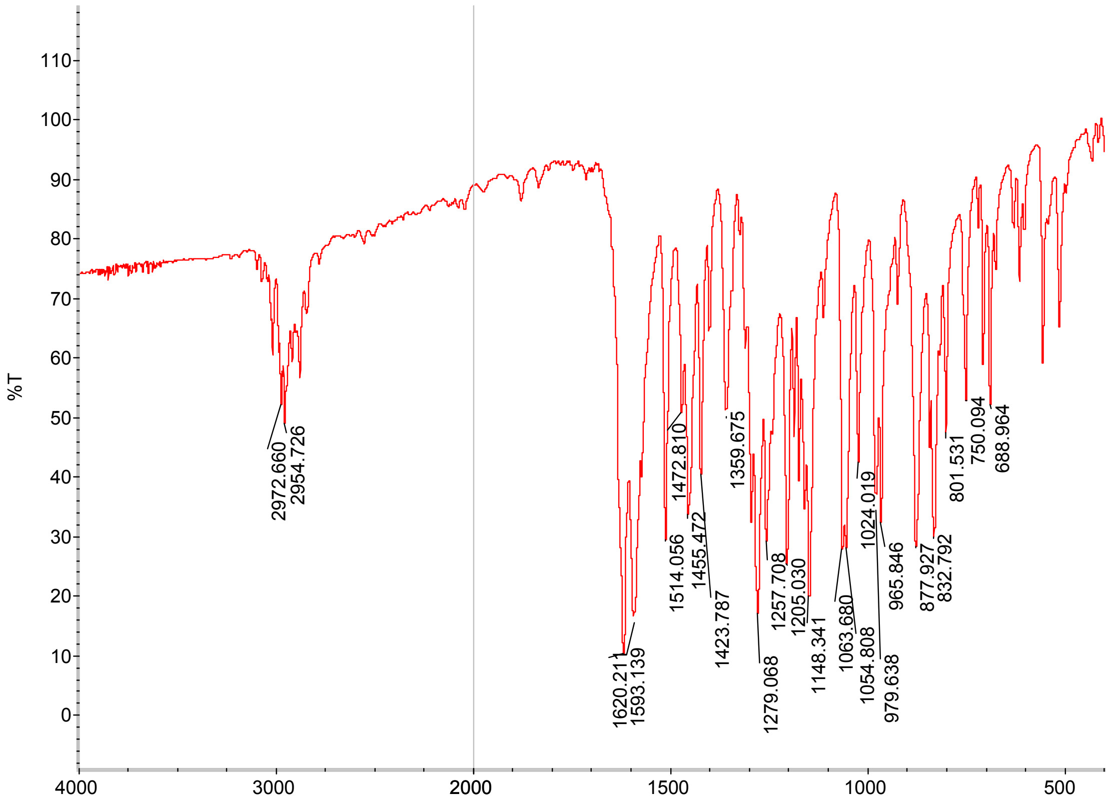

3c

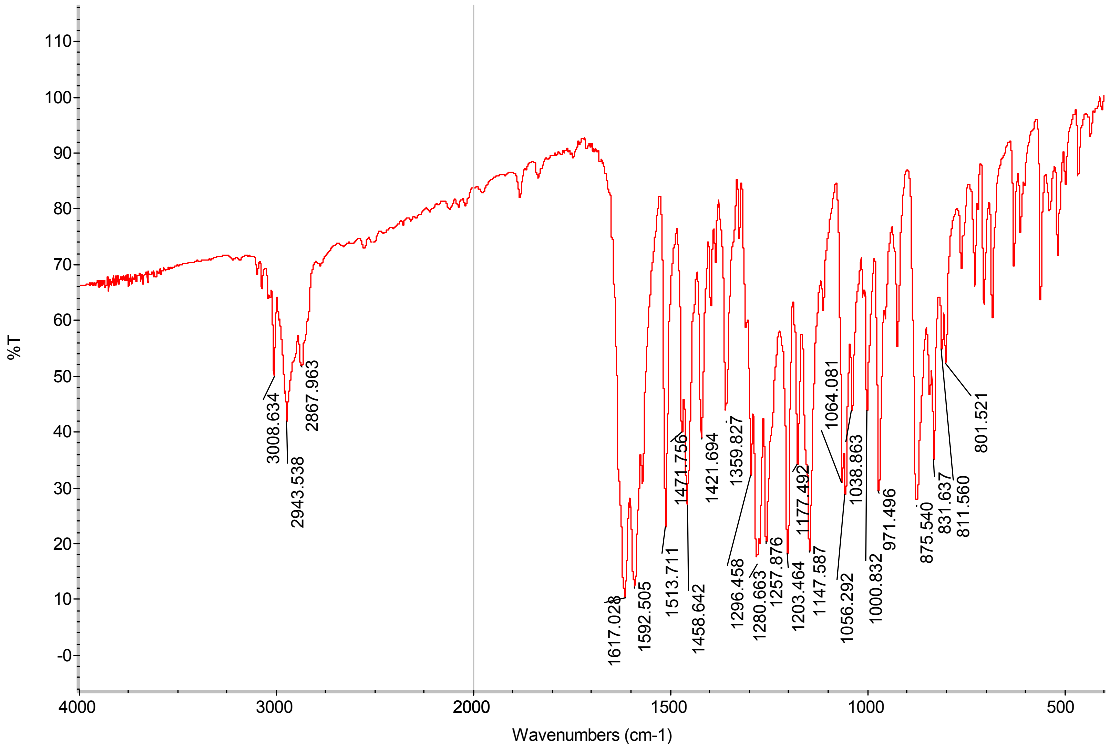
%T
Wavenumbers (cm-1)
3008.634
2943.538
2867.963
1617.028
1592.505
1513.711
1471.756
1458.642
1421.694
1359.827
1296.458
1280.663
1257.876
1203.464
1177.492
1147.587
1064.081
1056.292
1038.863
1000.832
971.496
875.540
831.637
811.560
801.521

3d

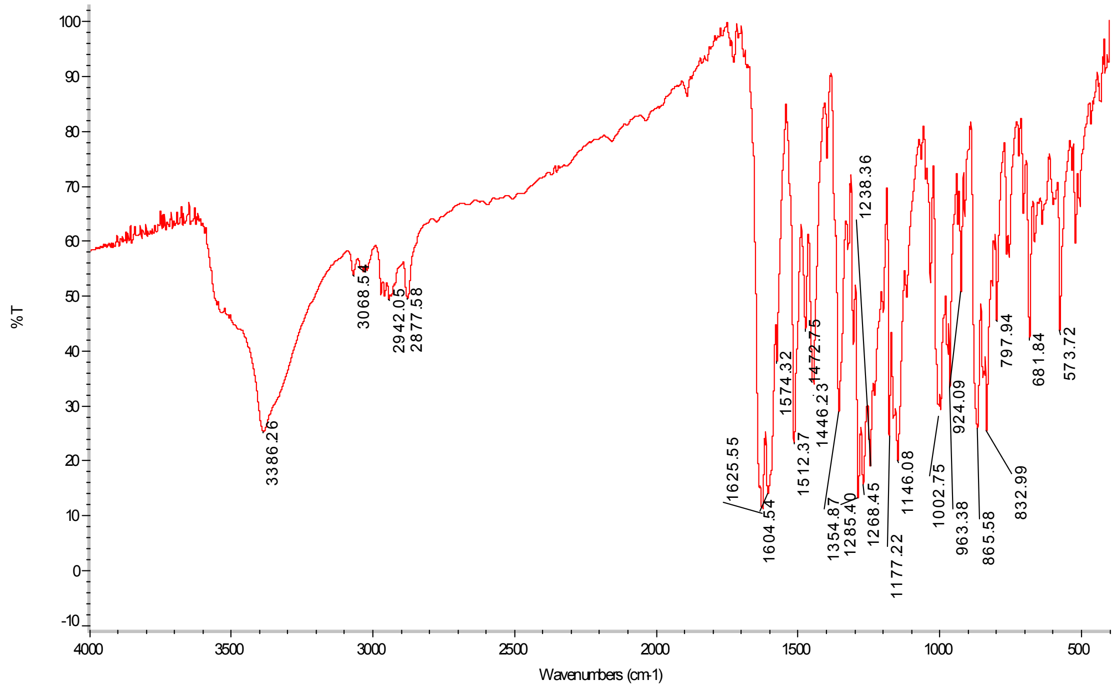
%T
Wavenumbers (cm-1)
3386.26
3068.54
2942.05
2877.58
1625.55
1604.54
1574.32
1512.37
1472.75
1446.23
1354.87
1285.40
1268.45
1238.36
1177.22
1146.08
1002.75
963.38
924.09
865.58
832.99
797.94
681.84
573.72

3e

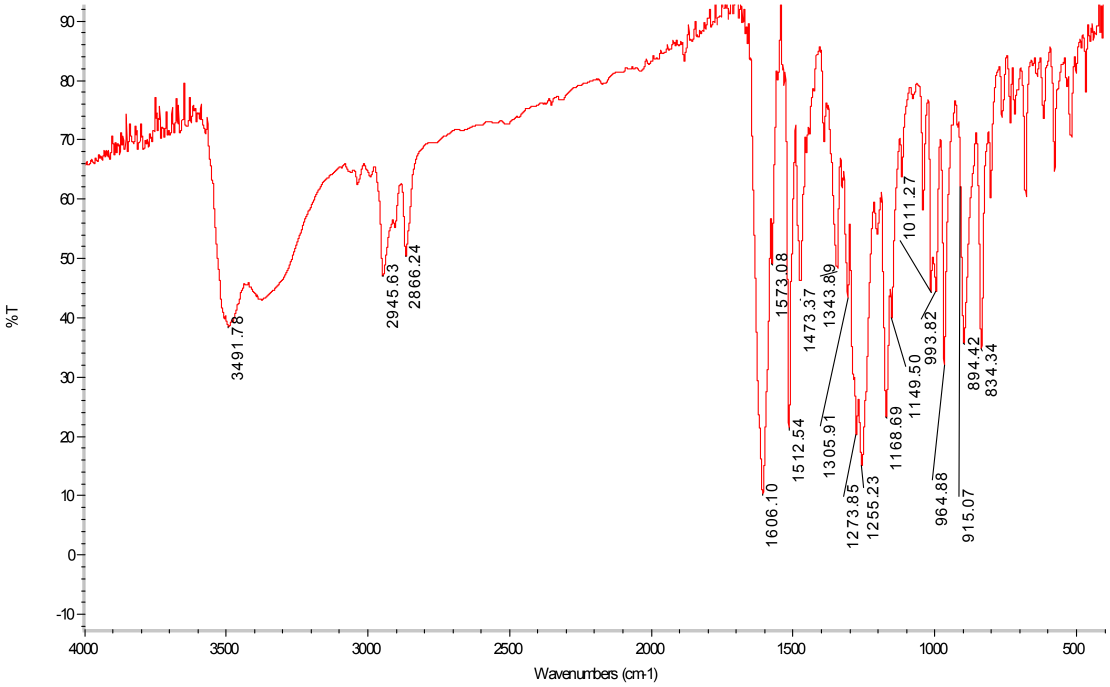
%T
Wavenumbers (cm-1)
3491.78
2945.63
2866.24
1606.10
1573.08
1512.54
1473.37
1343.89
1305.91
1273.85
1255.23
1168.69
1149.50
1011.27
993.82
964.88
915.07
894.42
834.34

7a

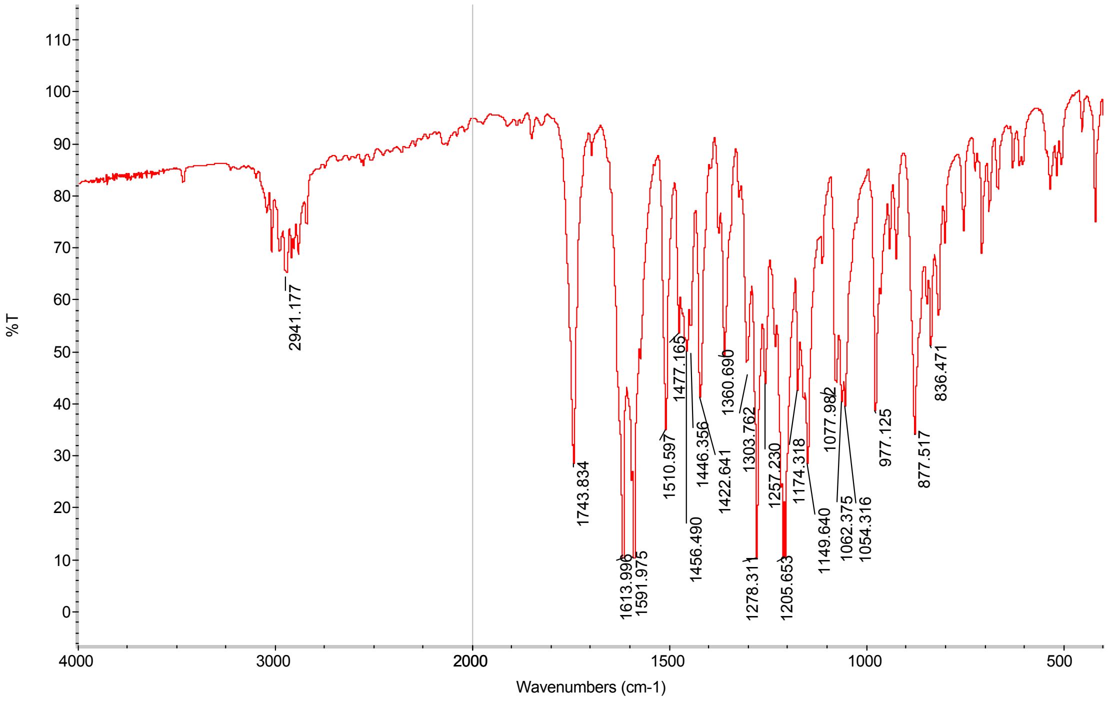
%T
Wavenumbers (cm-1)
2941.177
1743.834
1613.996
1591.975
1510.597
1477.165
1456.490
1446.356
1422.641
1360.690
1303.762
1278.311
1257.230
1205.653
1174.318
1149.640
1077.982
1062.375
1054.316
977.125
877.517
836.471

7b

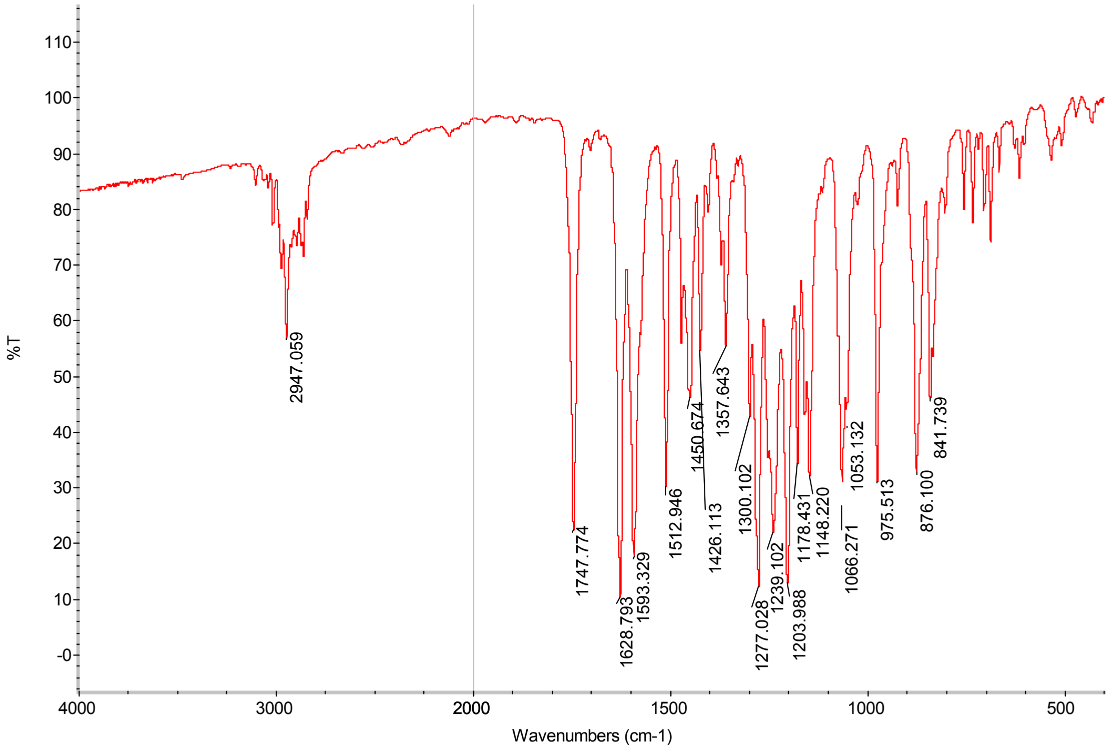
%T
Wavenumbers (cm-1)
2947.059
1747.774
1628.793
1593.329
1512.946
1450.674
1426.113
1357.643
1300.102
1277.028
1239.102
1203.988
1178.431
1148.220
1066.271
1053.132
975.513
876.100
841.739

7c

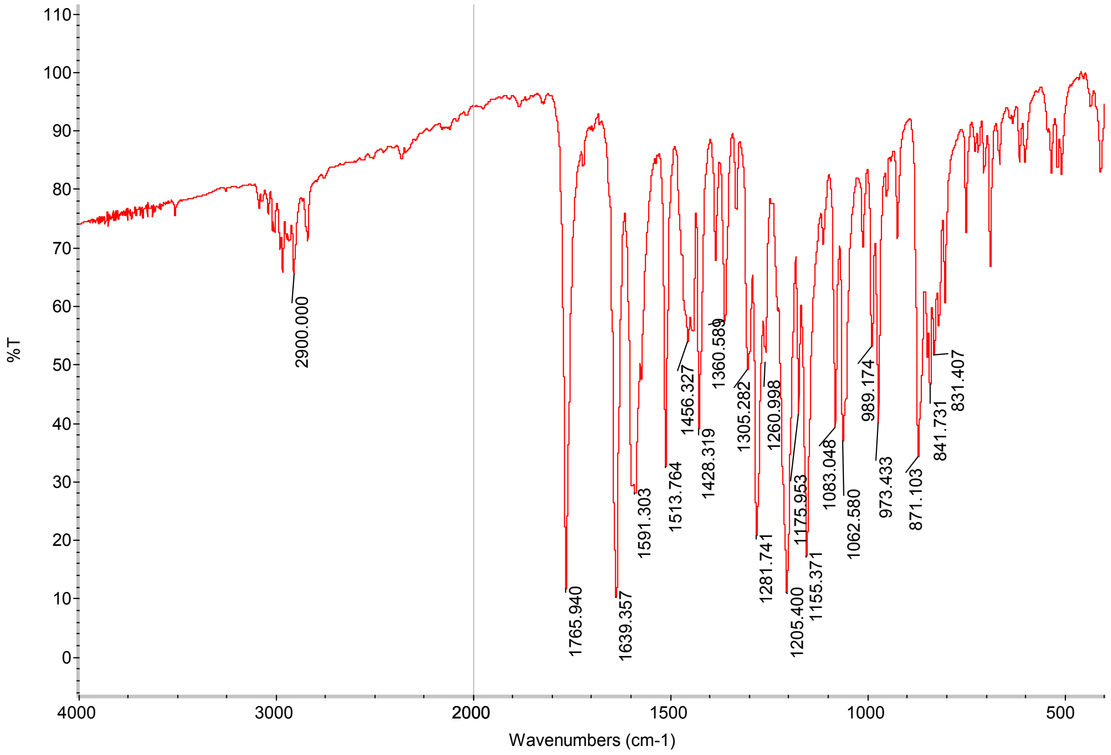
%T
Wavenumbers (cm-1)
2900.000
1765.940
1639.357
1591.303
1513.764
1456.327
1428.319
1360.589
1305.282
1281.741
1260.998
1205.400
1175.953
1155.371
1083.048
1062.580
989.174
973.433
871.103
841.731
831.407

HPLC spectra of compounds 3a-7c

Specify methods used: Instrument:Agilent 1100;

Column:Agilent SB-C18, 30 x 2.1 mm,3.5 um;

Detector:DAD detector, 254 nm;

Flow rate: 1 ml / min

Mobile phase: methanol: water (0.1% glacial acetic acid); gradient elution

Injection volume: 5 ul

Temperature: 25 ° C

Spectra without purity table means that the purity is close to 100%.

3a

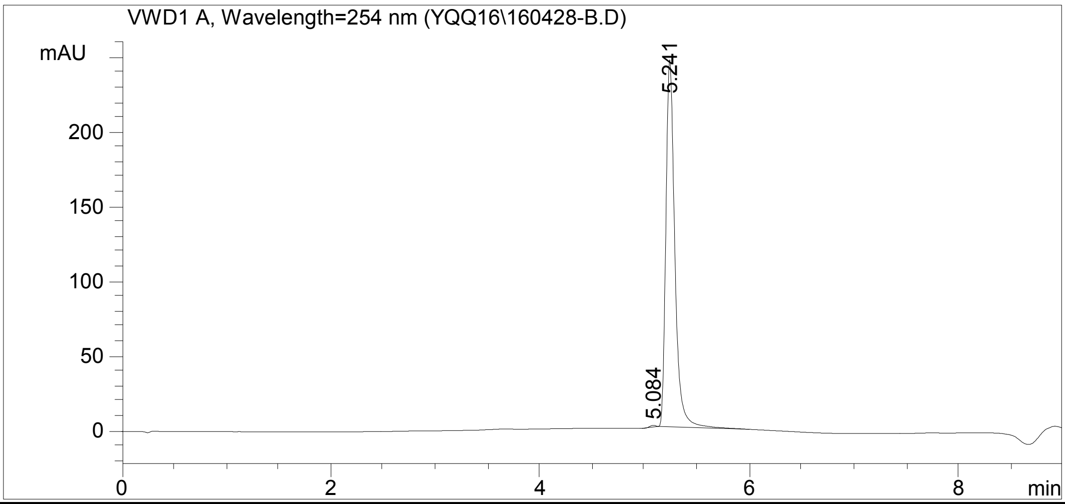

#	Meas. Ret. Time	Main Peak Heigh	Main Peak Area	Main Peak Area %
1	5.084	1.126	3.856	0.262
2	5.241	245.901	1464.061	99.737

3b

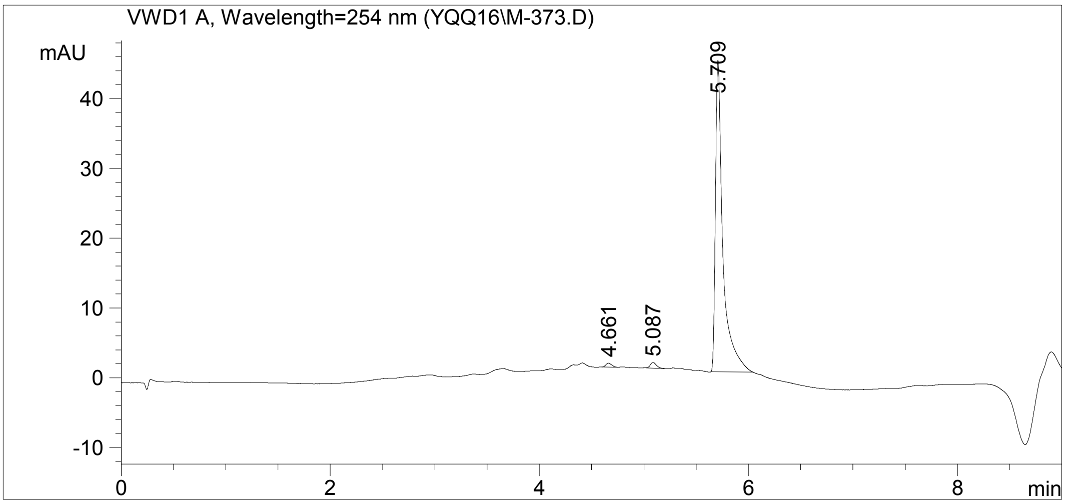

#	Meas. Ret. Time	Main Peak Heigh	Main Peak Area	Main Peak Area %
1	4.661	0.570	2.222	0.973
2	5.087	0.830	3.364	1.472
3	5.709	44.707	222.807	97.554

3c

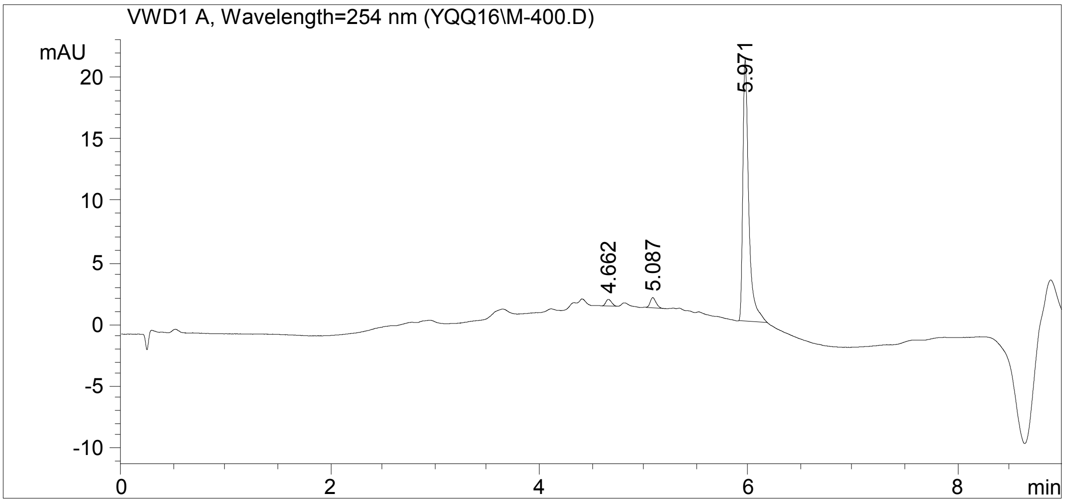

#	Meas. Ret. Time	Main Peak Heigh	Main Peak Area	Main Peak Area %
1	4.662	0.570	2.222	0.973
2	5.087	0.830	3.364	1.472
3	5.971	44.707	222.807	97.554

3d

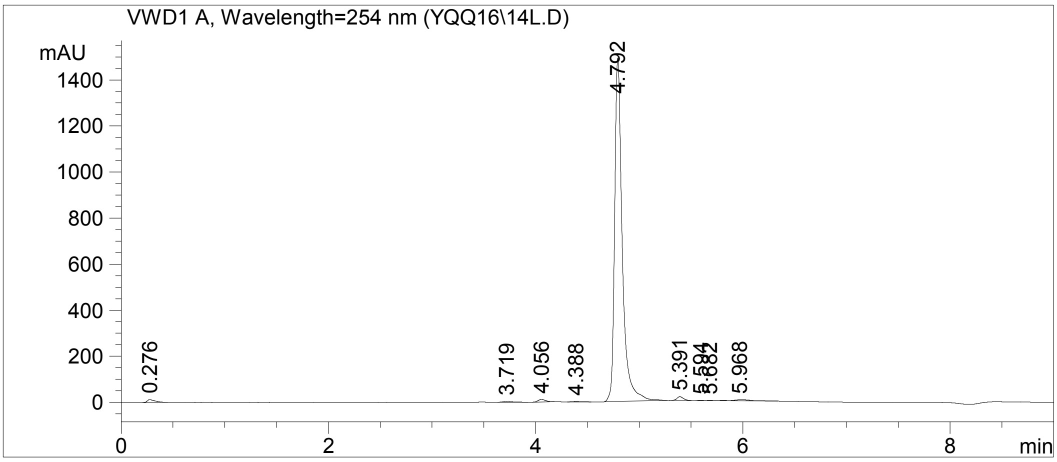

#	Meas. Ret. Time	Main Peak Heigh	Main Peak Area	Main Peak Area %
1	0.276	12.486	63.275	0.794
2	3.719	3.917	25.006	0.313
3	4.056	11.271	56.760	0.712
4	4.388	2.031	11.980	0.150
5	4.792	1492.323	7663.139	96.196
6	5.391	16.904	73.912	0.927
7	5.594	0.744	1.940	0.024
8	5.682	1.145	3.323	0.041
9	5.968	4.525	66.782	0.838

3e

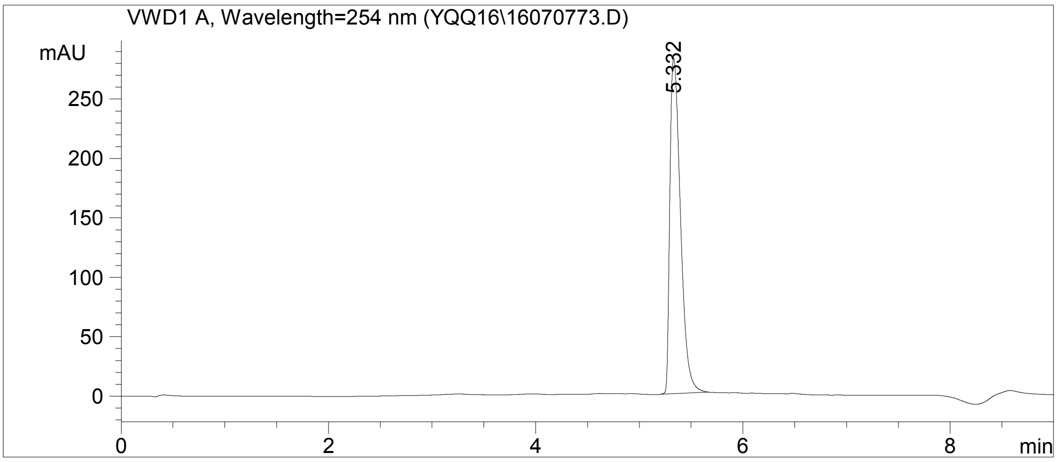

This means that the purity is close to 100%.

7a

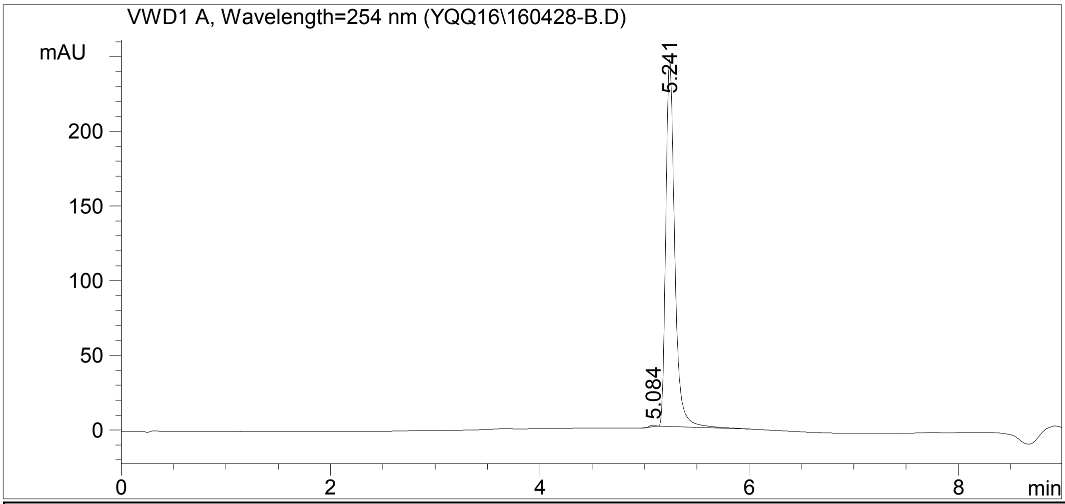

#	Meas. Ret. Time	Main Peak Heigh	Main Peak Area	Main Peak Area %
1	5.084	1.126	3.856	0.262
2	5.241	245.901	1464.061	99.737

7b

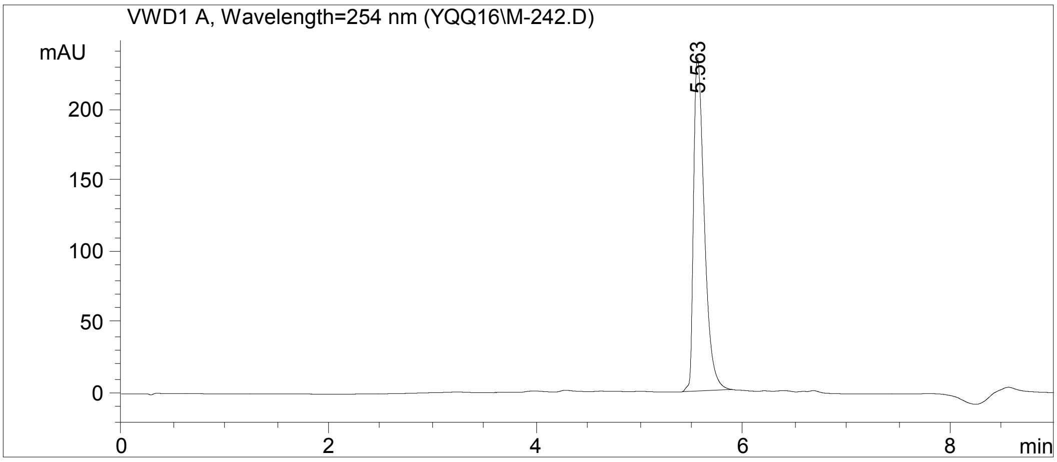

This means that the purity is close to 100%.

7c

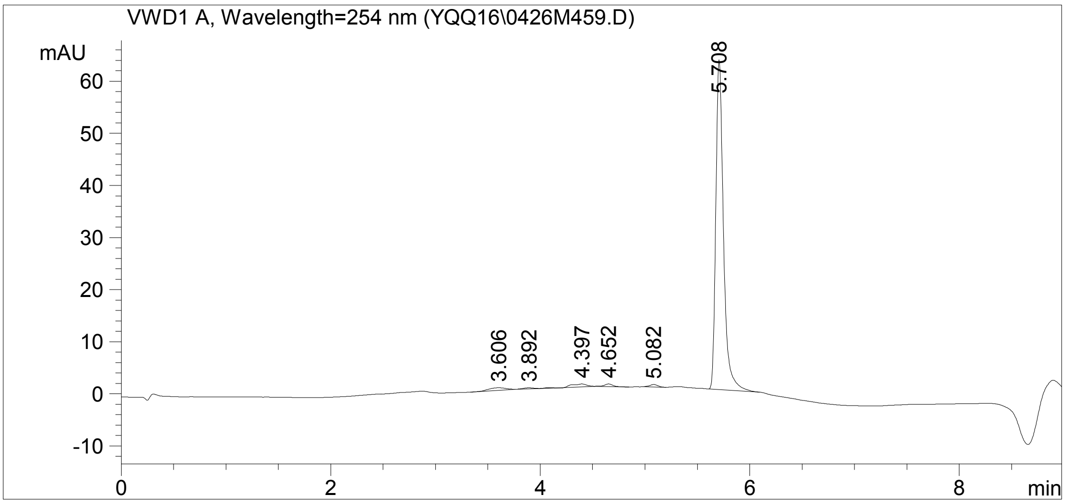

#	Meas. Ret. Time	Main Peak Heigh	Main Peak Area	Main Peak Area %
1	3.606	0.486	5.667	1.667
2	3.892	0.198	1.271	0.373
3	4.397	0.536	5.568	1.638
4	4.652	0.504	2.979	0.876
5	5.082	0.474	2.570	0.756
6	5.708	63.319	321.843	94.688

Mass spectrum of compounds 3a-7c

3a

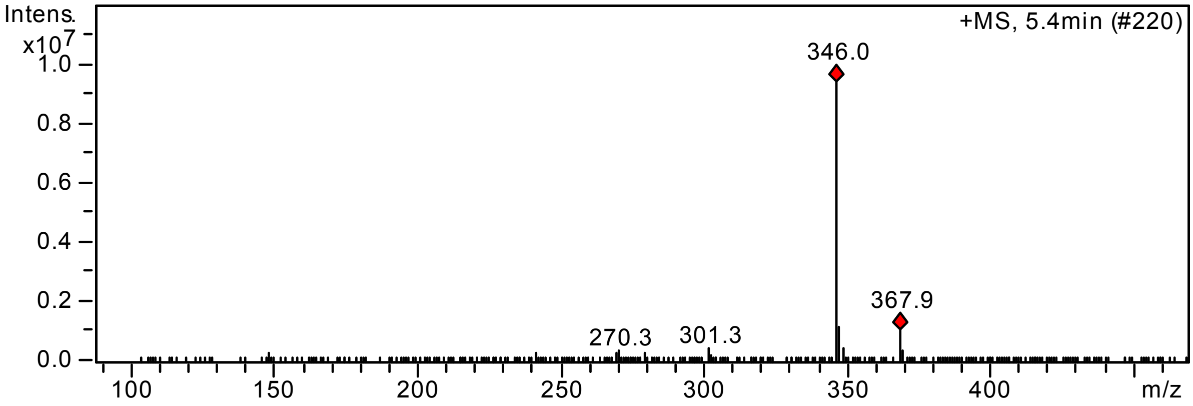

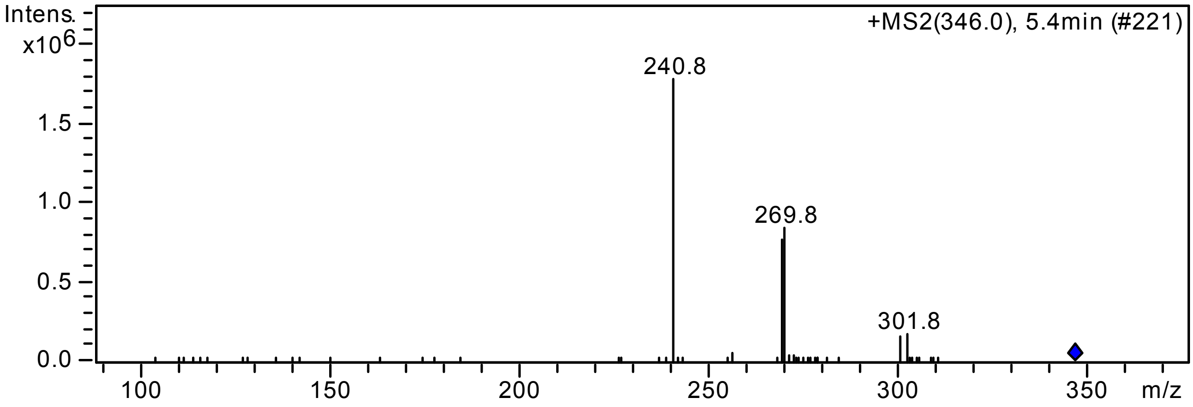

3b

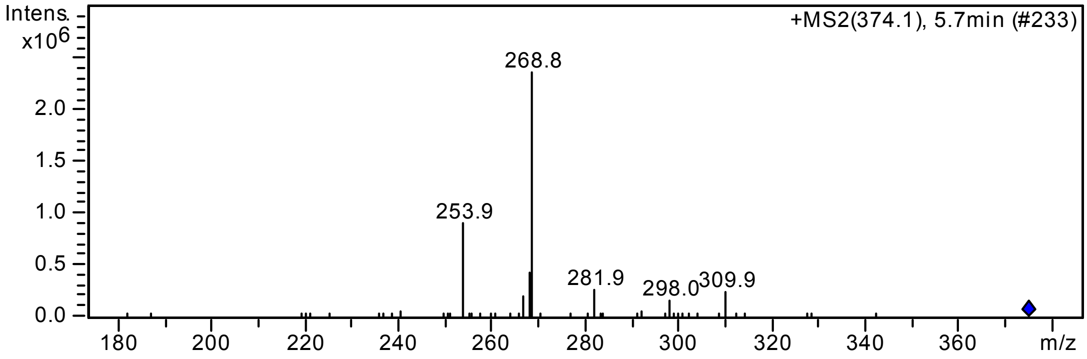

3c

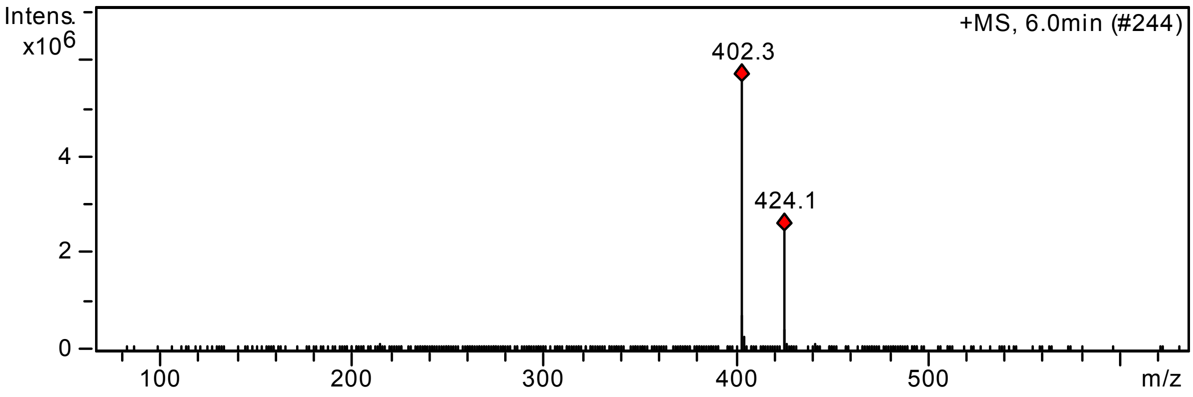

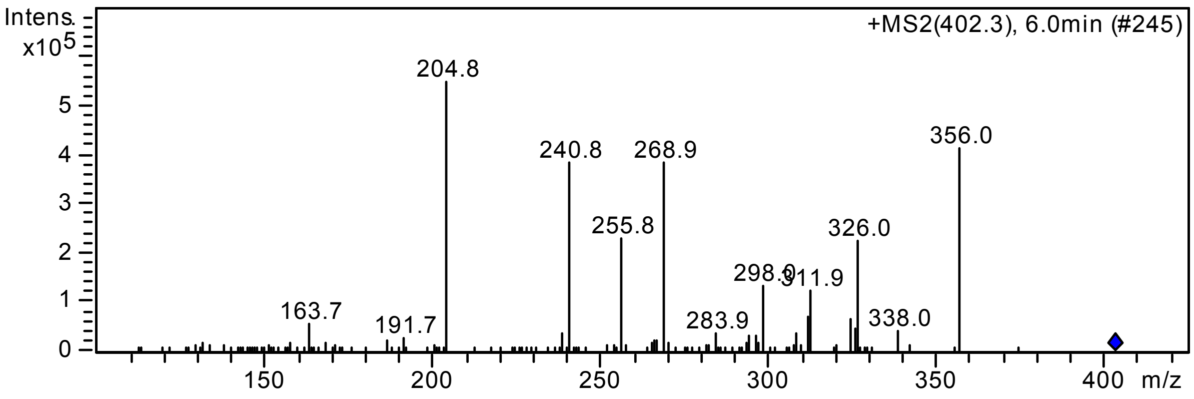
Intens.
x10^5
+MS2(402.3), 6.0min (#245)
204.8
240.8
268.9
356.0
255.8
326.0
163.7
191.7
283.9
338.0
m/z

3d

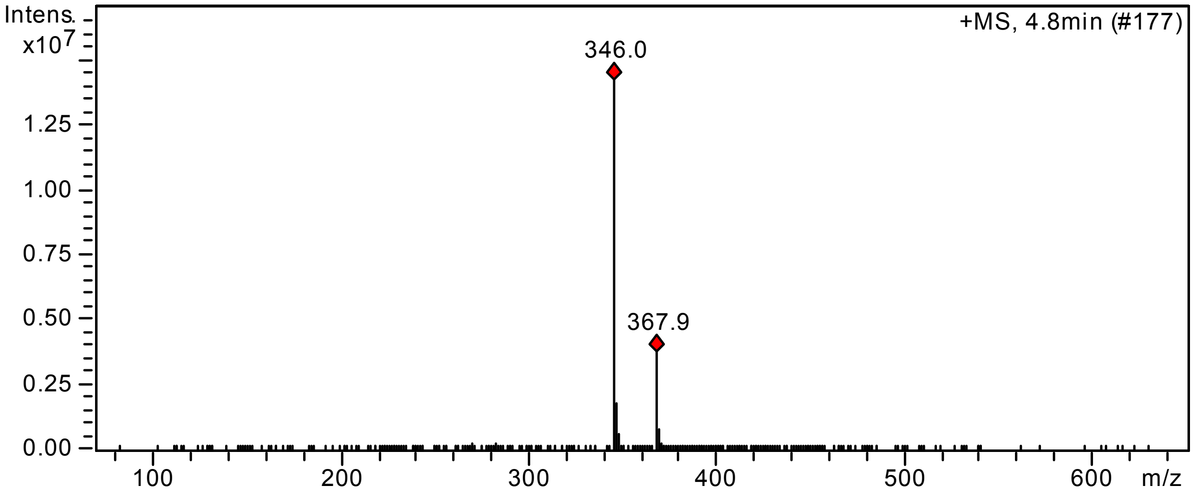
Intens.
x10^7
+MS, 4.8min (#177)
346.0
367.9
m/z

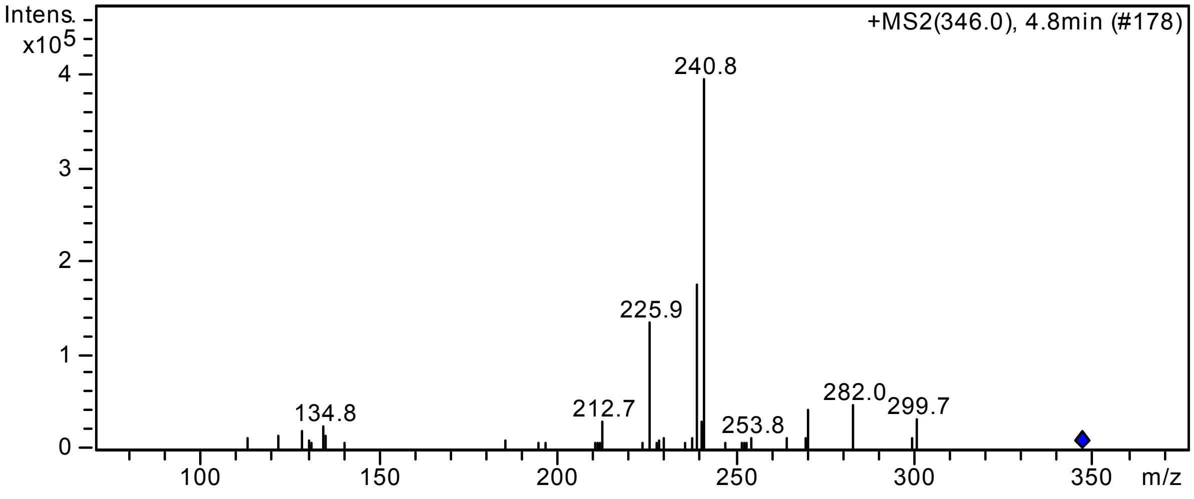
Intens.
x10^5
+MS2(346.0), 4.8min (#178)
240.8
225.9
134.8
212.7
253.8
282.0
299.7
m/z

3e

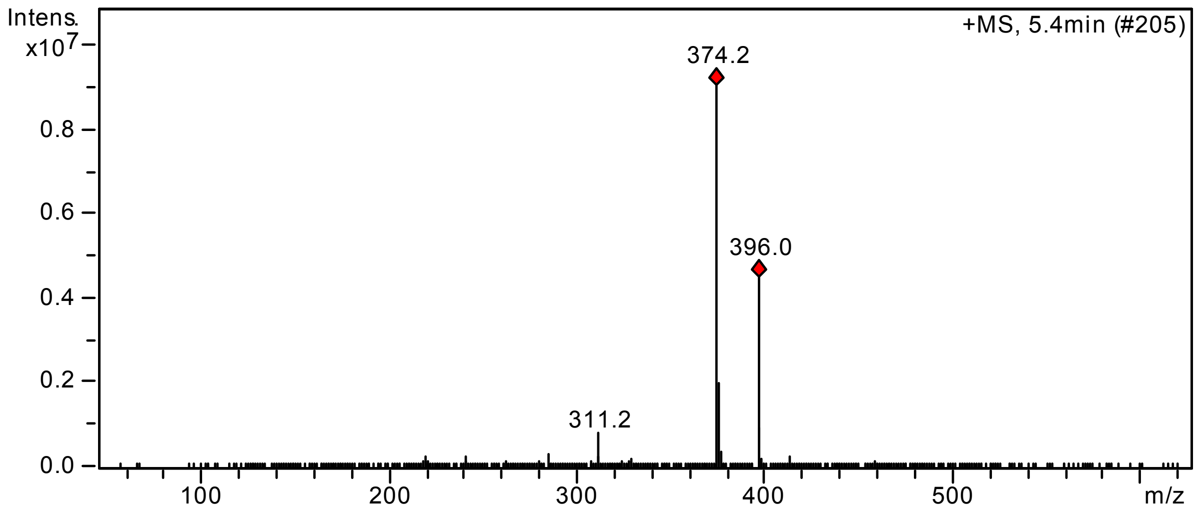
Intens.
x10^7
+MS, 5.4min (#205)
374.2
396.0
311.2
0.0
0.2
0.4
0.6
0.8
100
200
300
400
500
m/z

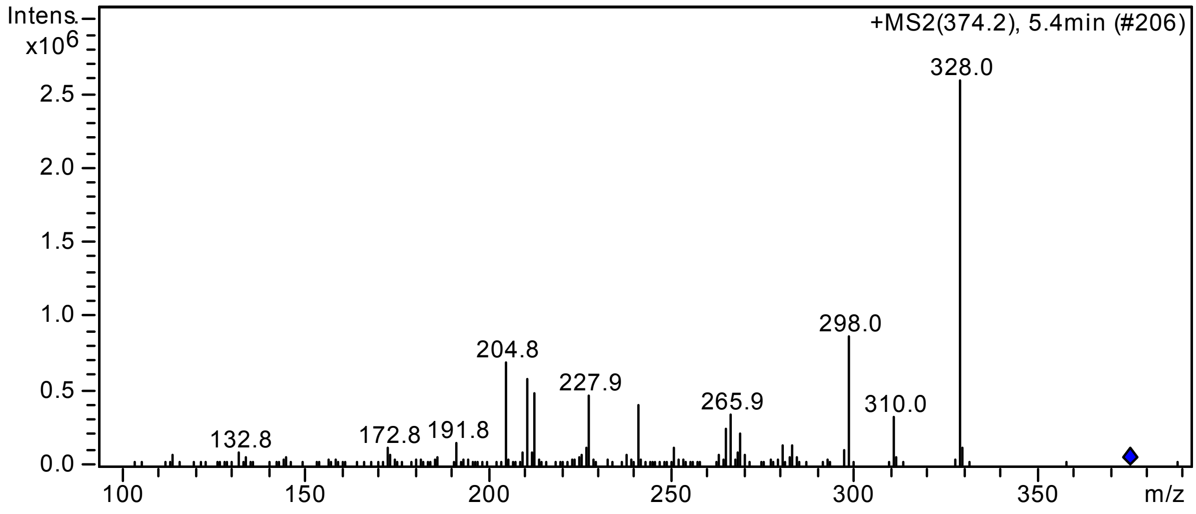
Intens.
x10^6
+MS2(374.2), 5.4min (#206)
328.0
298.0
204.8
227.9
265.9
310.0
132.8
172.8
191.8
0.0
0.5
1.0
1.5
2.0
2.5
100
150
200
250
300
350
m/z

7a

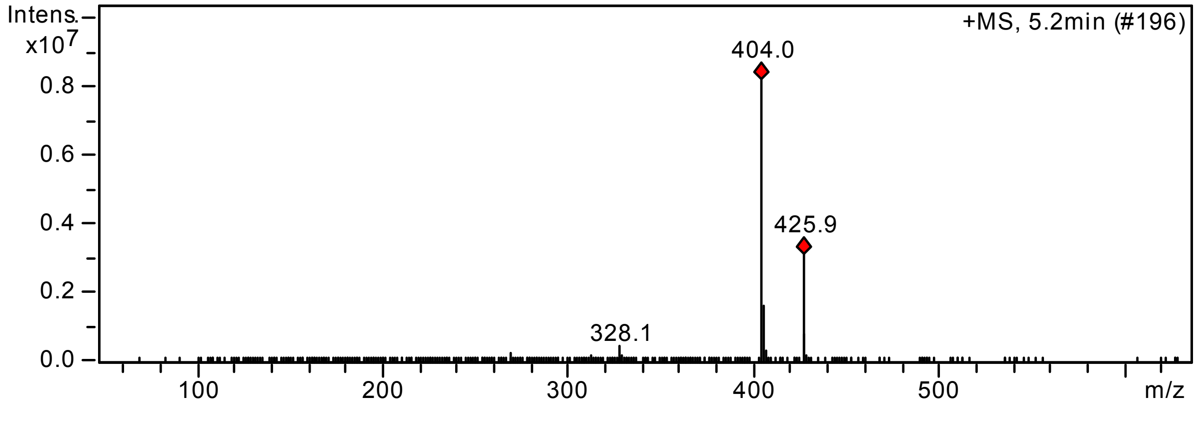
Intens.
x10^7
+MS, 5.2min (#196)
404.0
425.9
328.1
0.0
0.2
0.4
0.6
0.8
100
200
300
400
500
m/z

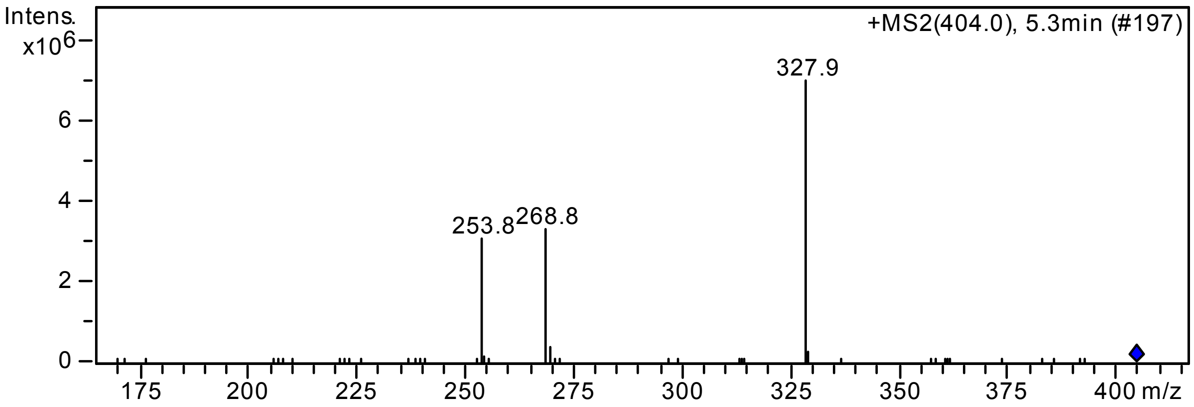
Intens.
x10^6
+MS2(404.0), 5.3min (#197)
327.9
253.8
268.8
m/z

7b

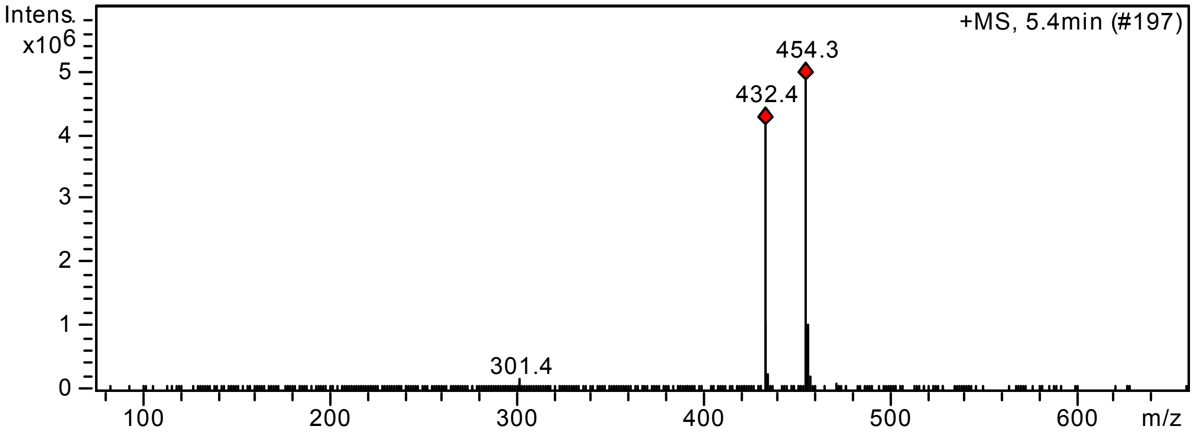
Intens.
x10^6
+MS, 5.4min (#197)
454.3
432.4
301.4
m/z

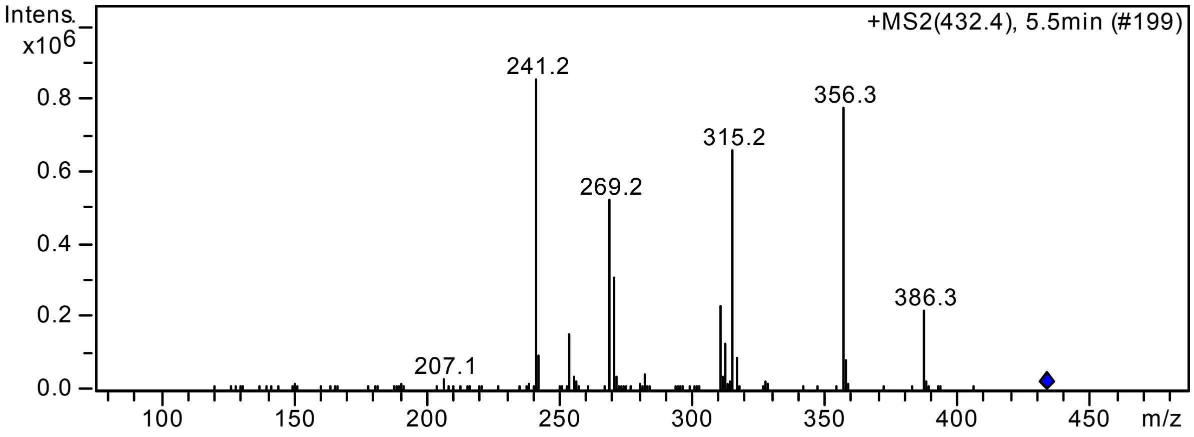
Intens.
x10^6
+MS2(432.4), 5.5min (#199)
241.2
356.3
315.2
269.2
386.3
207.1
m/z

7c

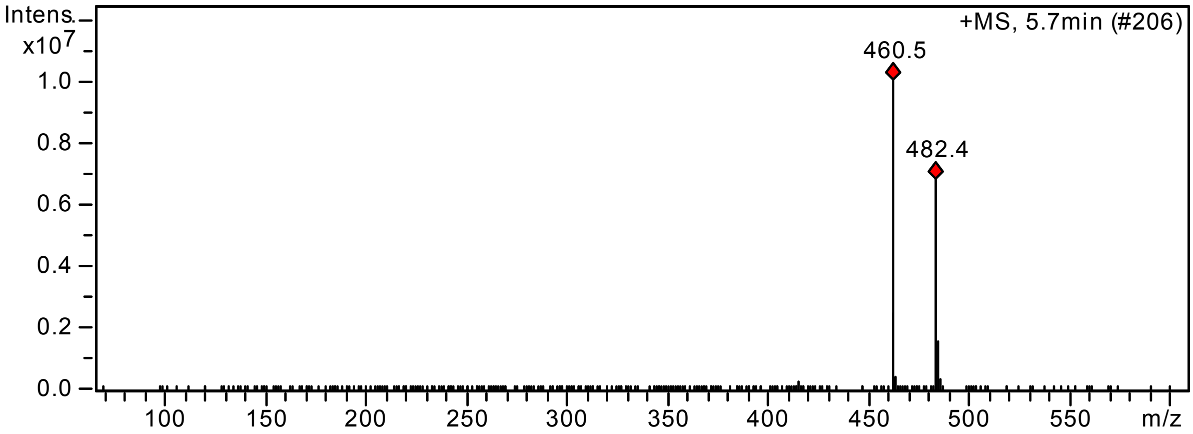
Intens.
x10^7
+MS, 5.7min (#206)
460.5
482.4
1.0
0.8
0.6
0.4
0.2
0.0
100
150
200
250
300
350
400
450
500
550
m/z

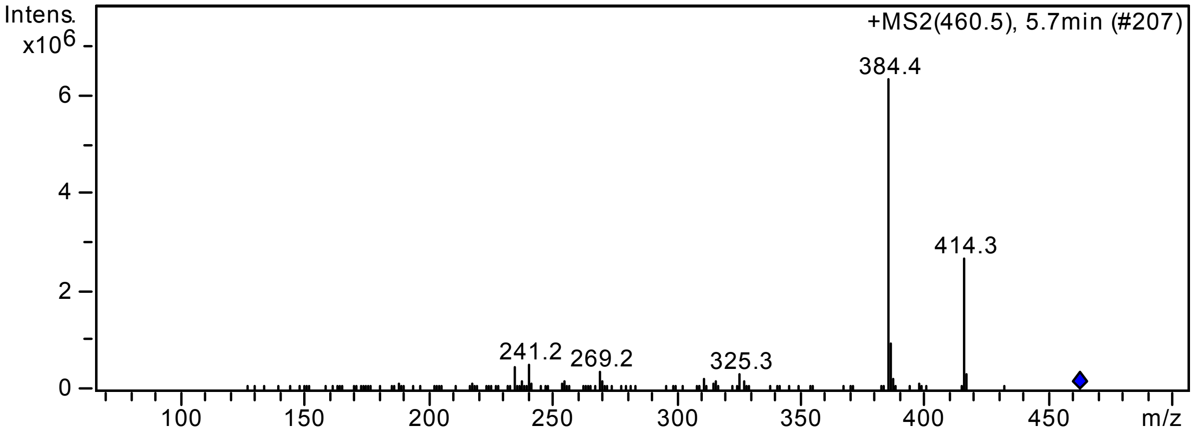
Intens.
x10^6
+MS2(460.5), 5.7min (#207)
384.4
414.3
241.2
269.2
325.3
6
4
2
0
100
150
200
250
300
350
400
450
m/z

^{1}H NMR(600 MHz, DMSO-d$_6$) of compounds 3a-7c

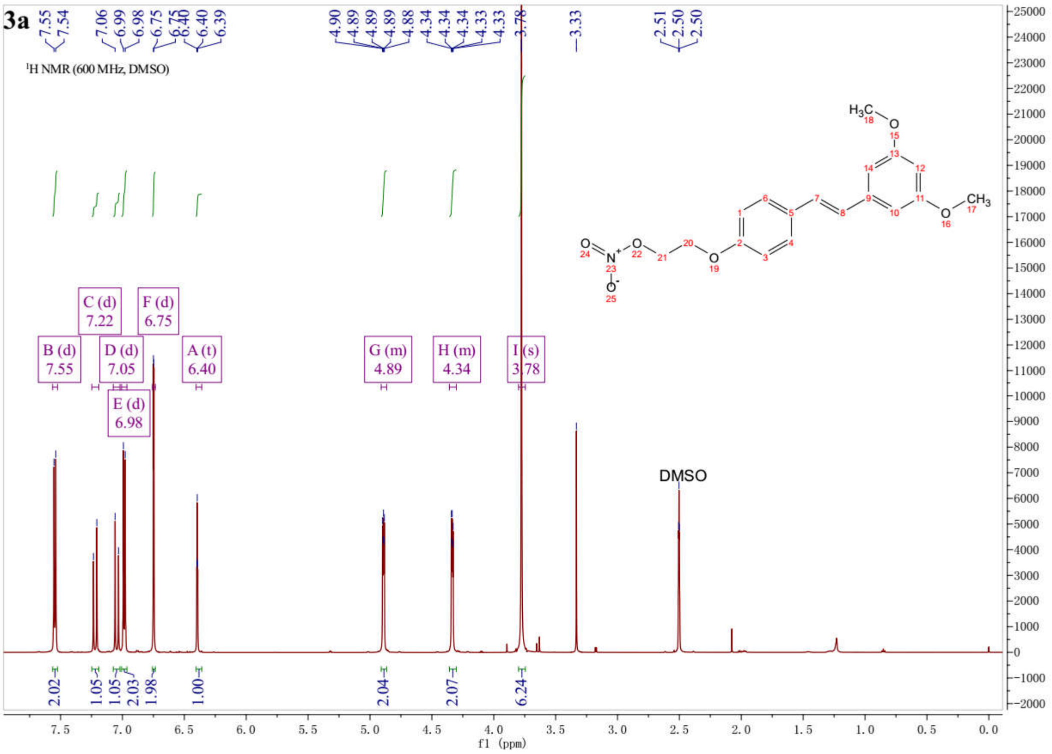

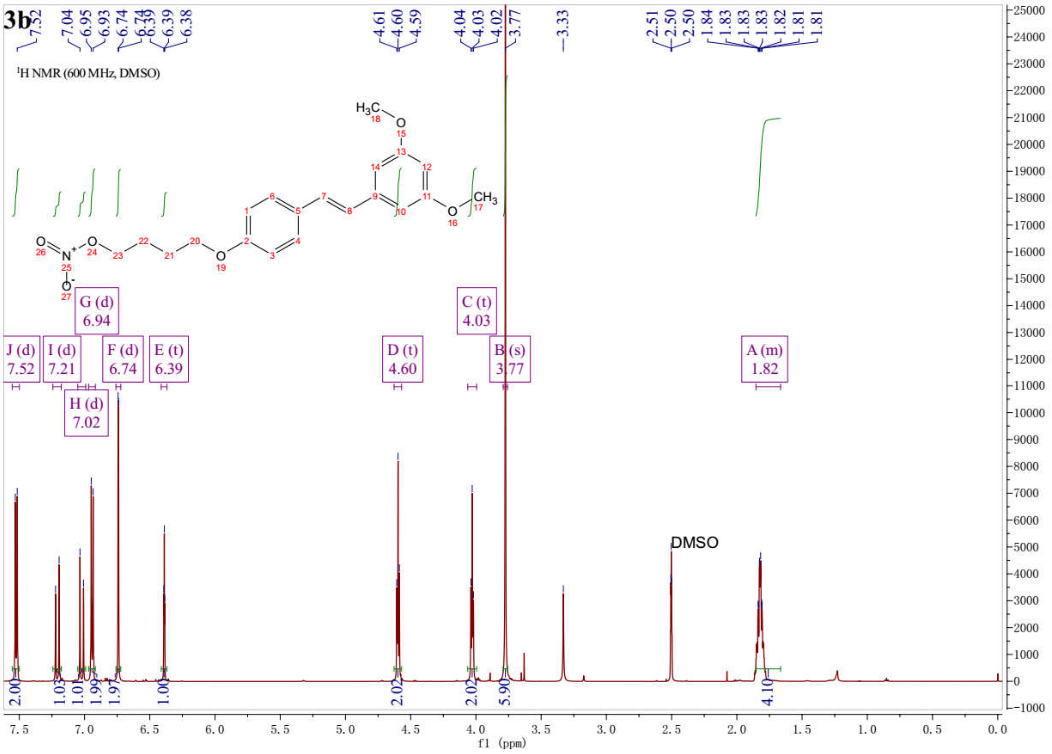

3c

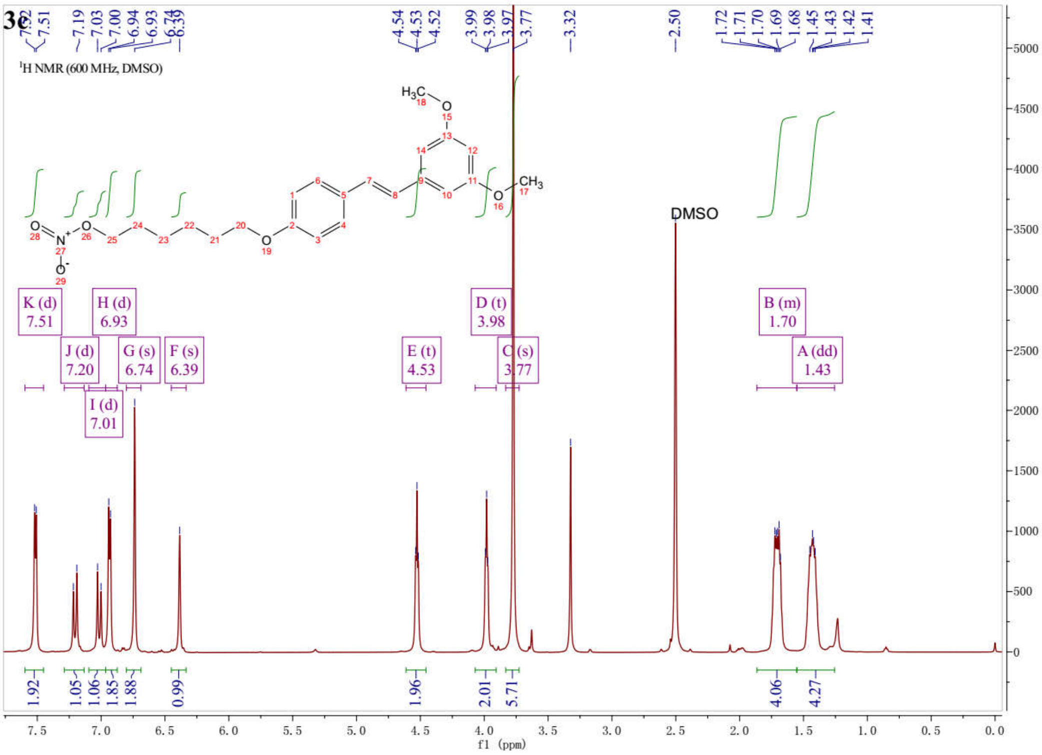

¹H NMR (600 MHz, DMSO)
DMSO
K (d) 7.51
J (d) 7.20
I (d) 7.01
H (d) 6.93
G (s) 6.74
F (s) 6.39
E (t) 4.53
D (t) 3.98
C (s) 3.77
B (m) 1.70
A (dd) 1.43
f1 (ppm)

3d

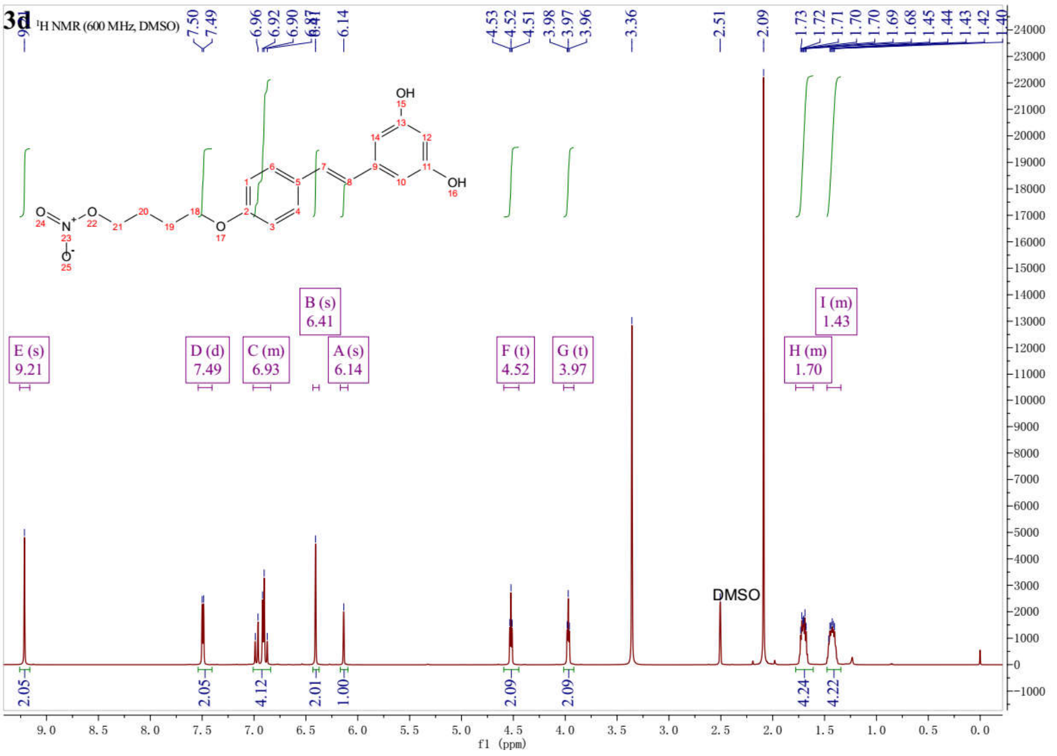

¹H NMR (600 MHz, DMSO)
DMSO
E (s) 9.21
D (d) 7.49
C (m) 6.93
B (s) 6.41
A (s) 6.14
F (t) 4.52
G (t) 3.97
H (m) 1.70
I (m) 1.43
f1 (ppm)

3e

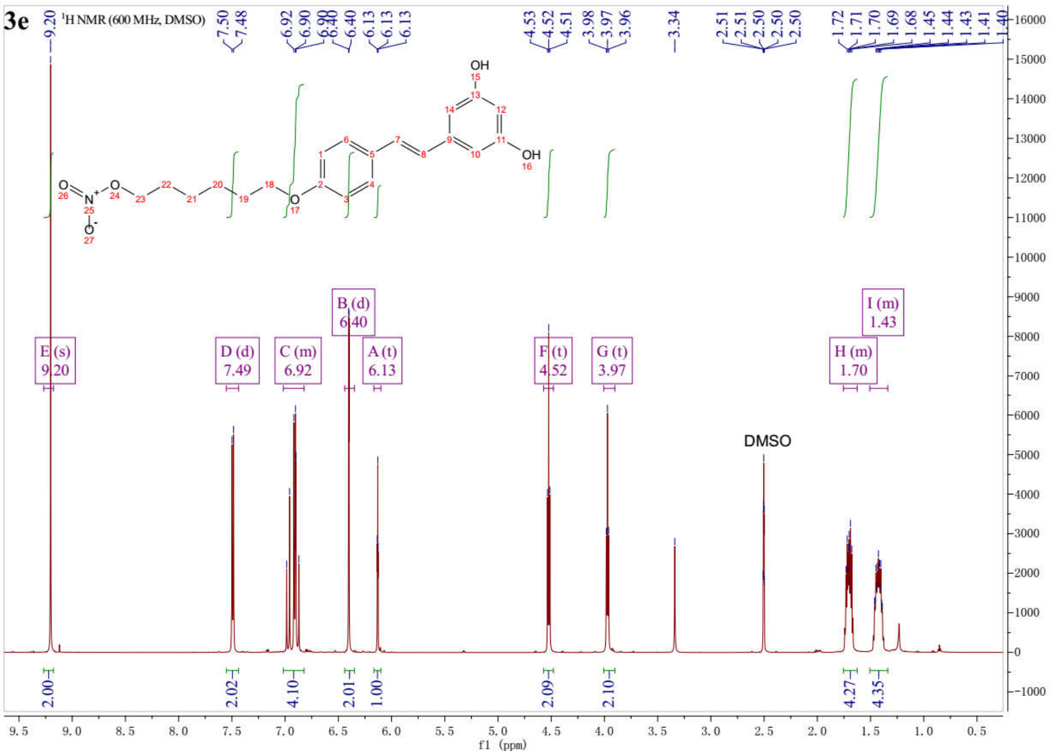

¹H NMR (600 MHz, DMSO)
E (s) 9.20
D (d) 7.49
C (m) 6.92
B (d) 6.40
A (t) 6.13
F (t) 4.52
G (t) 3.97
H (m) 1.70
I (m) 1.43
DMSO
f1 (ppm)

7a

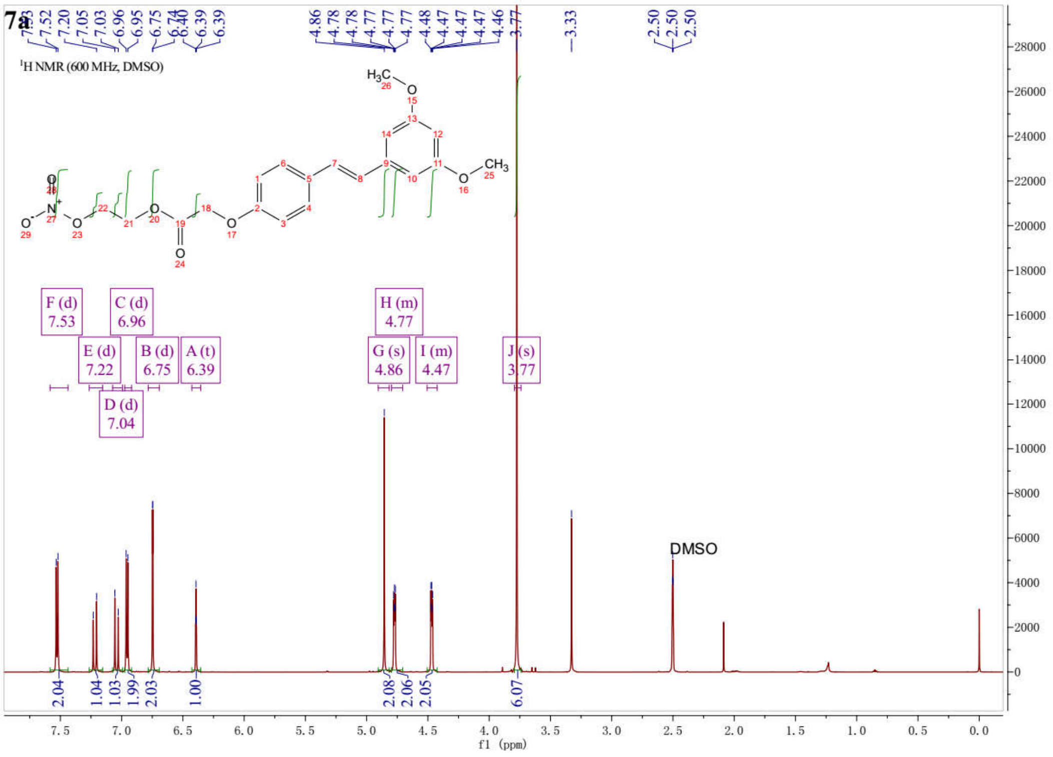

¹H NMR (600 MHz, DMSO)
F (d) 7.53
C (d) 6.96
E (d) 7.22
B (d) 6.75
A (t) 6.39
D (d) 7.04
H (m) 4.77
G (s) 4.86
I (m) 4.47
J (s) 3.77
DMSO
f1 (ppm)

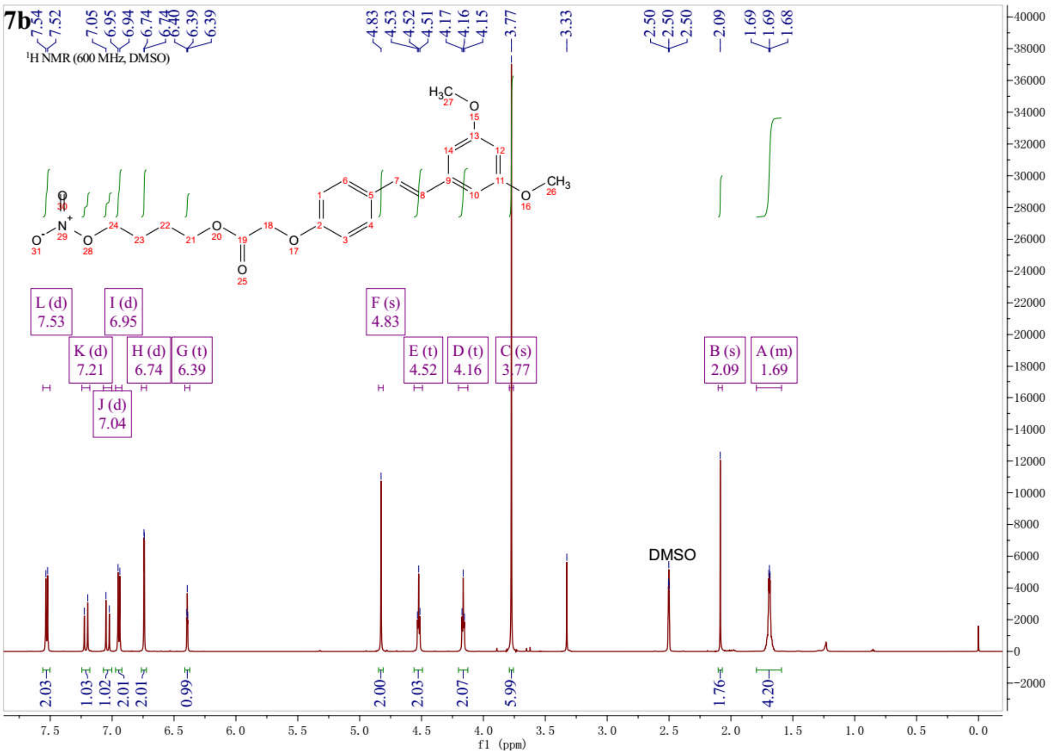
7b
¹H NMR (600 MHz, DMSO)
DMSO
f1 (ppm)

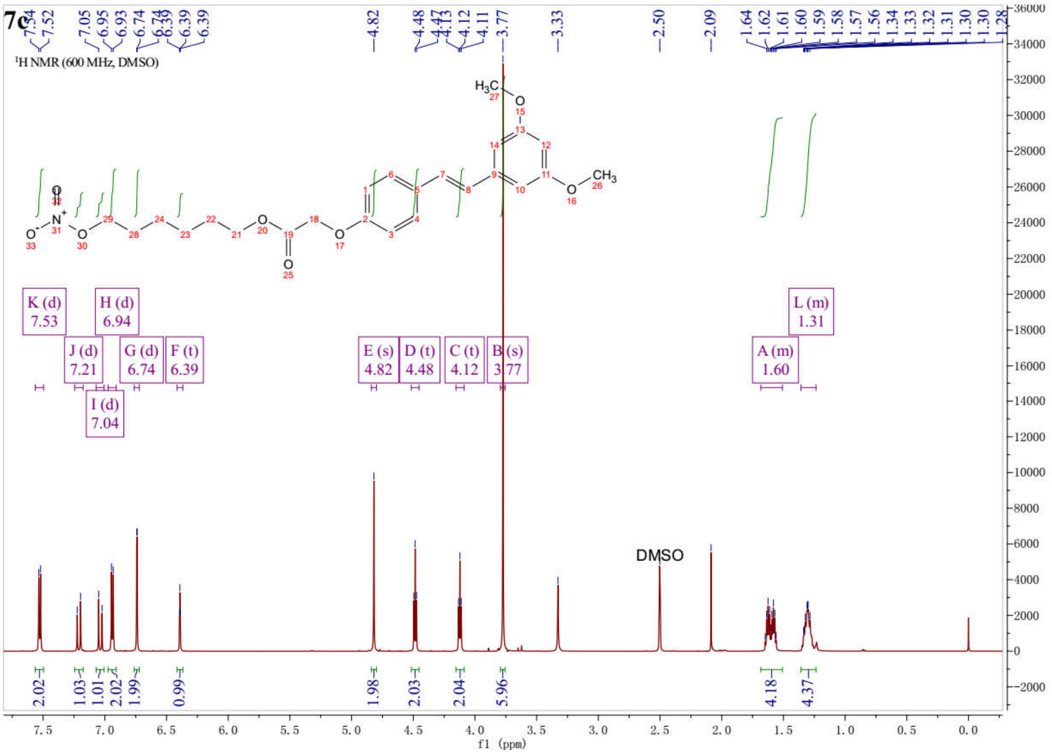
7c
¹H NMR (600 MHz, DMSO)
DMSO
f1 (ppm)

^{13}C NMR(150 MHz, DMSO-d_6) of compounds 3a-7c

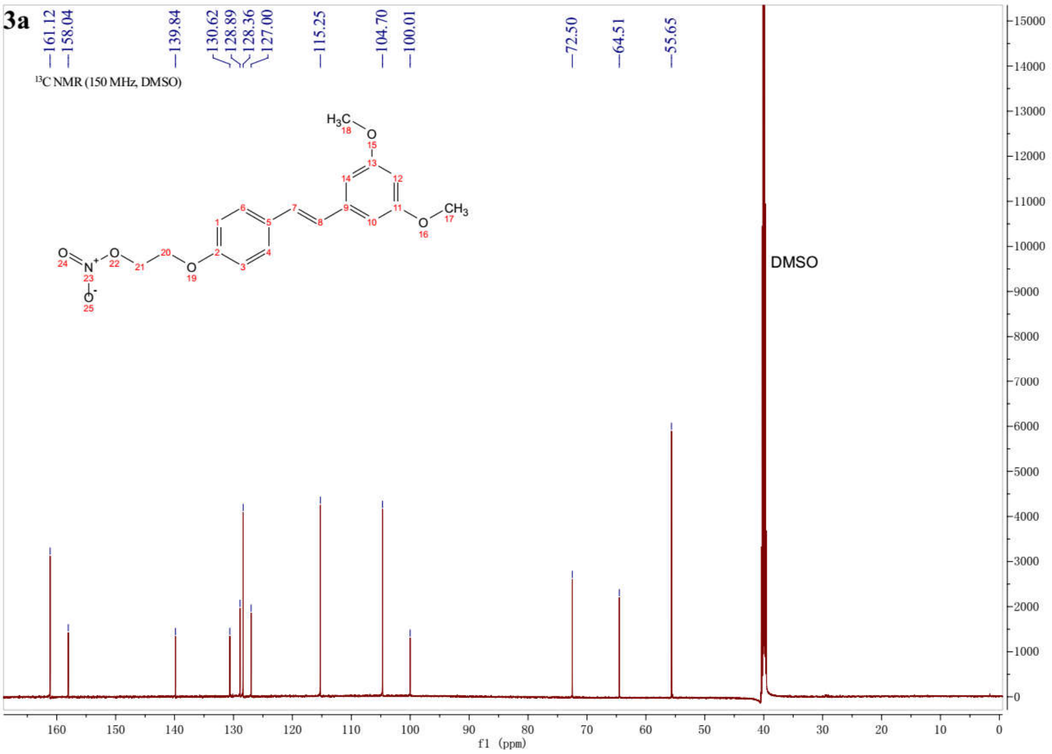

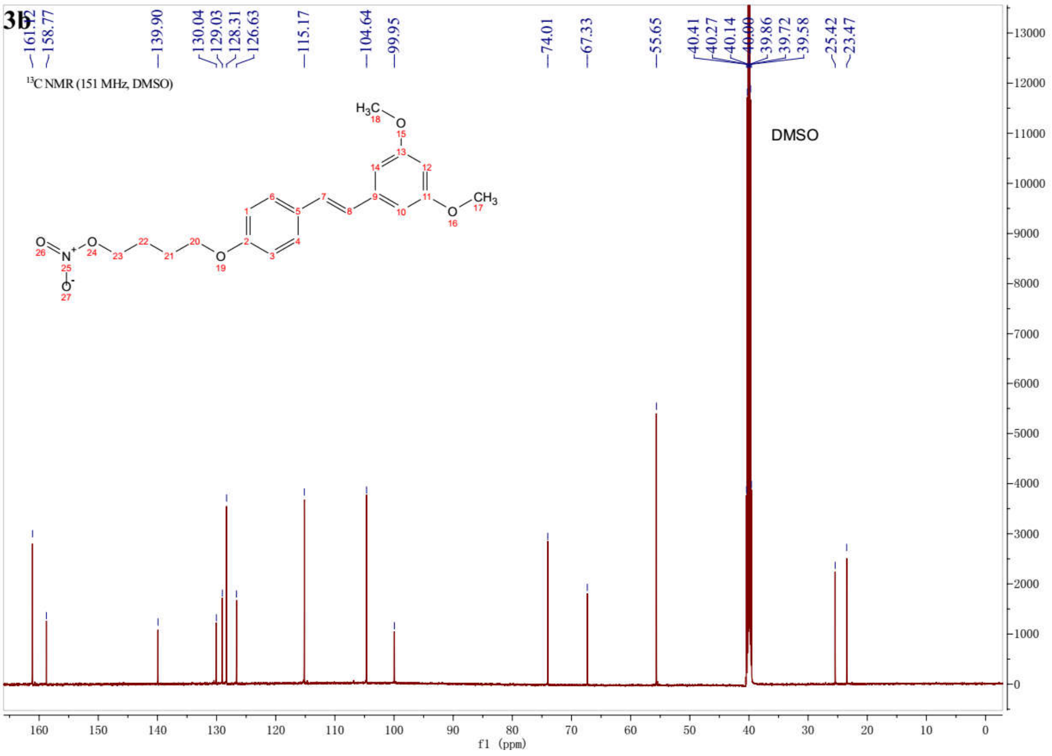

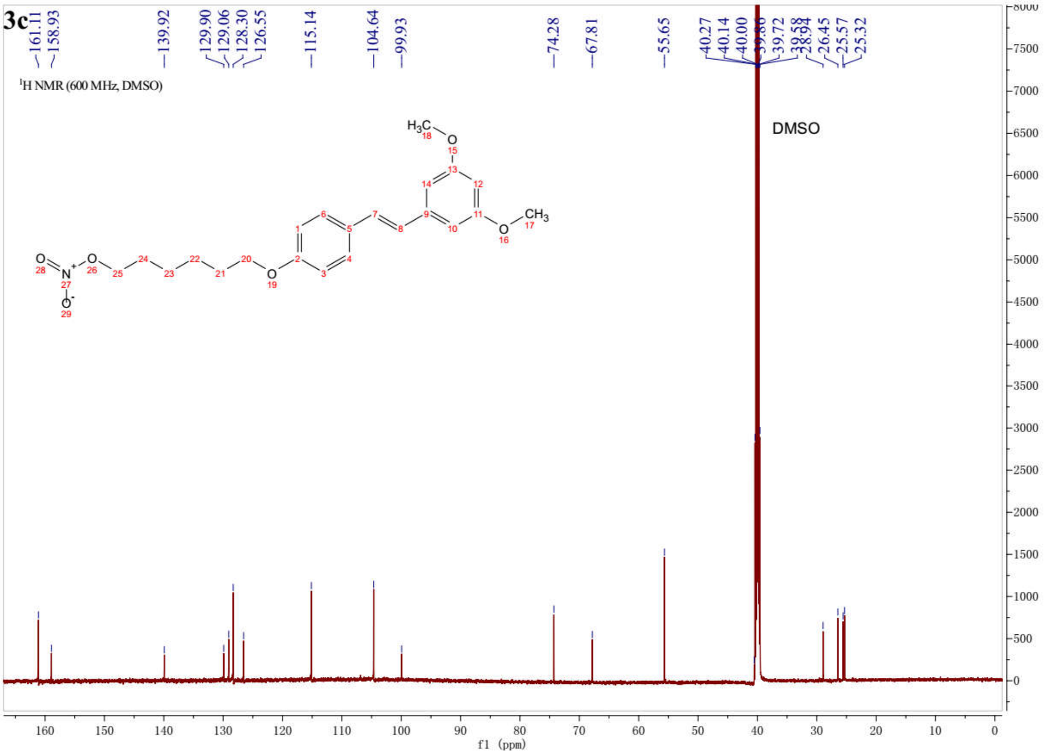
3c
161.11
158.93
139.92
129.90
129.06
128.30
126.55
115.14
104.64
99.93
74.28
67.81
55.65
40.27
40.14
40.00
39.72
26.45
25.57
25.32
1H NMR (600 MHz, DMSO)
DMSO
f1 (ppm)

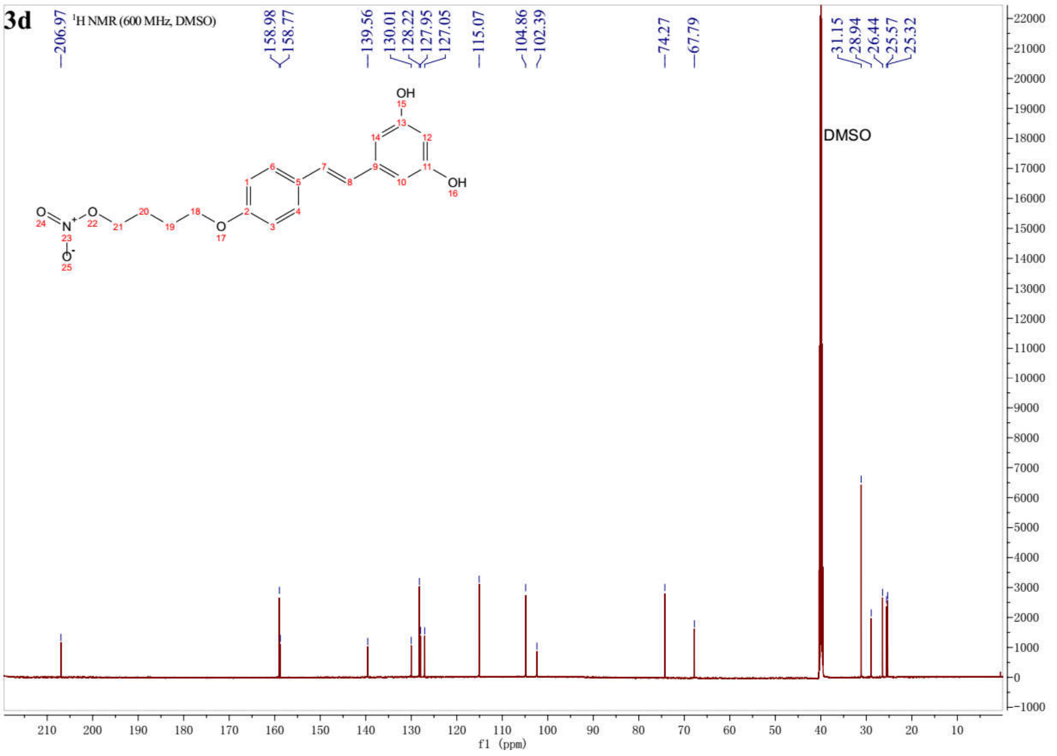
3d
206.97
1H NMR (600 MHz, DMSO)
158.98
158.77
139.56
130.01
128.22
127.95
127.05
115.07
104.86
102.39
74.27
67.79
31.15
28.94
26.44
25.57
25.32
DMSO
f1 (ppm)

3e

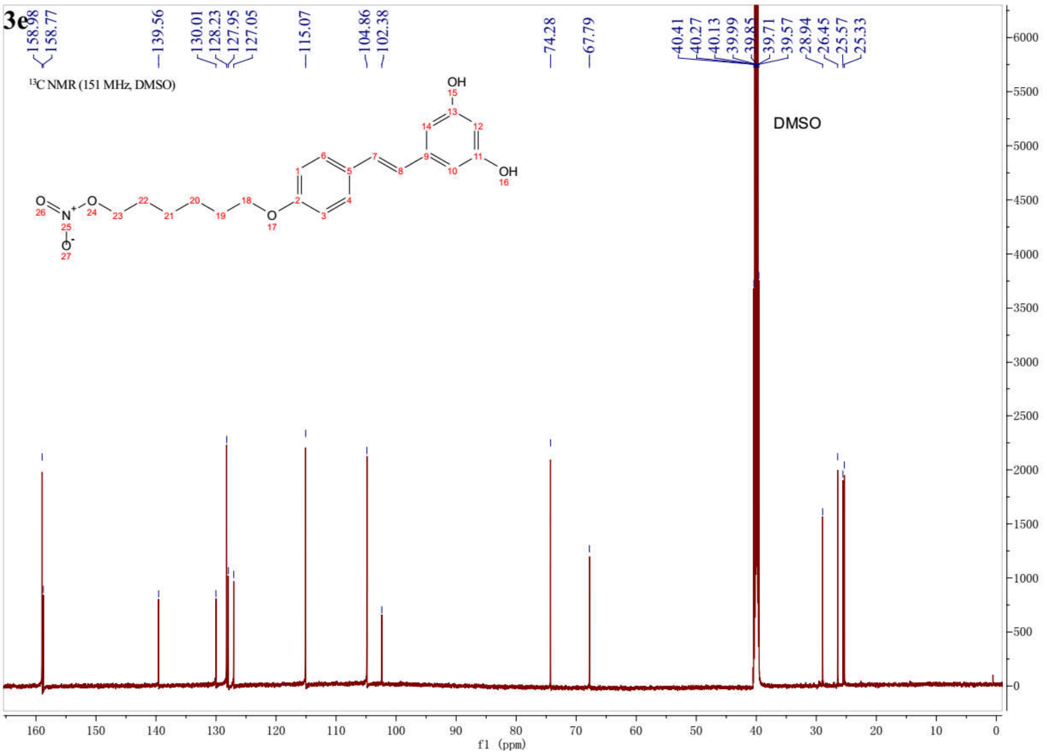
158.98
158.77
139.56
130.01
128.23
127.95
127.05
115.07
104.86
102.38
74.28
67.79
40.41
40.27
40.13
39.99
39.85
39.71
39.57
28.94
26.45
25.57
25.33
^{13}C NMR (151 MHz, DMSO)
DMSO
f1 (ppm)

7a

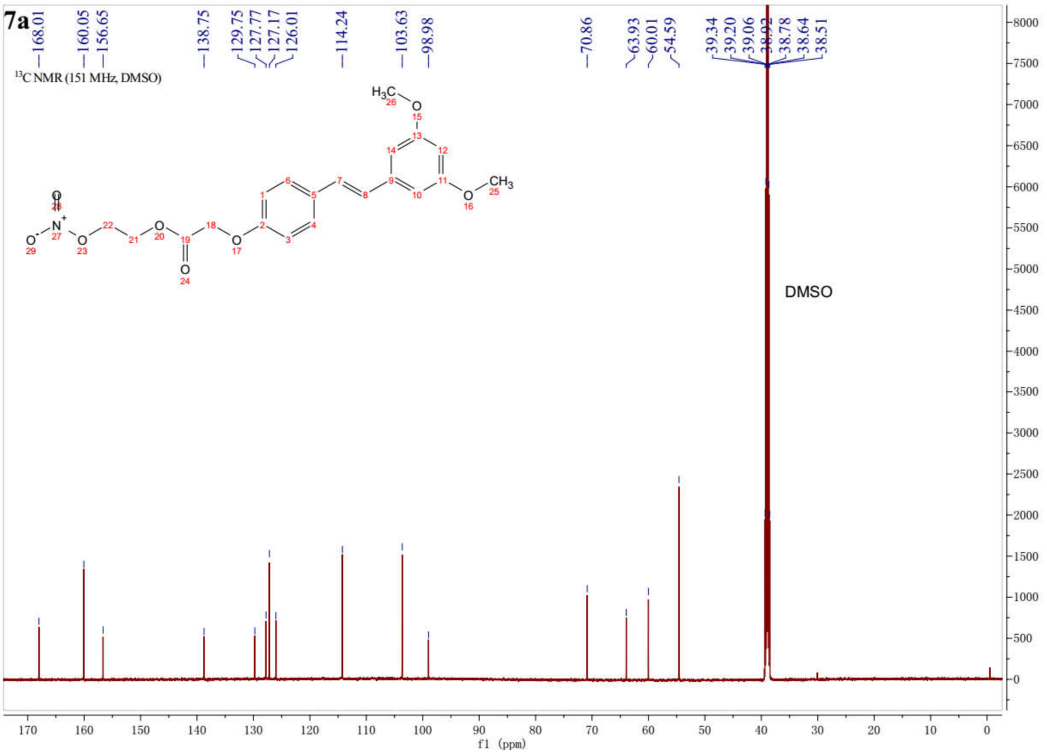
168.01
160.05
156.65
138.75
129.75
127.77
127.17
126.01
114.24
103.63
98.98
70.86
63.93
60.01
54.59
39.34
39.20
39.06
38.92
38.78
38.64
38.51
^{13}C NMR (151 MHz, DMSO)
DMSO
f1 (ppm)

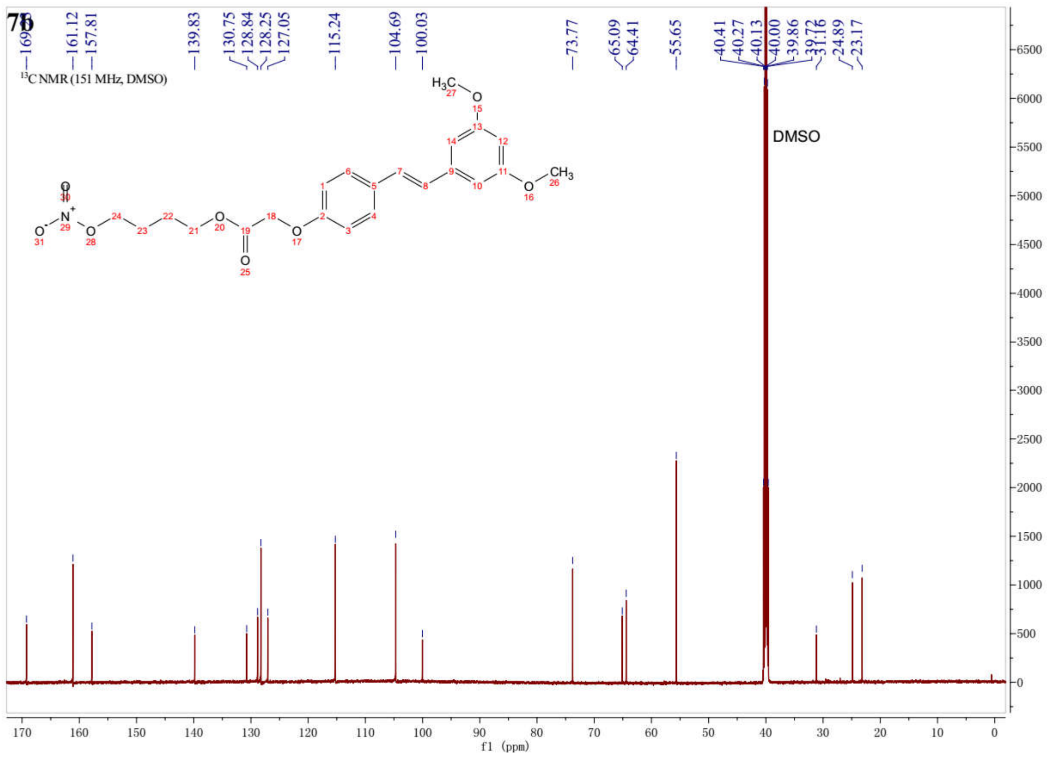
7b
^{13}C NMR (151 MHz, DMSO)
161.12
157.81
139.83
130.75
128.84
128.25
127.05
115.24
104.69
100.03
73.77
65.09
64.41
55.65
40.41
40.27
40.13
40.00
39.86
39.72
31.16
24.89
23.17
DMSO
f1 (ppm)

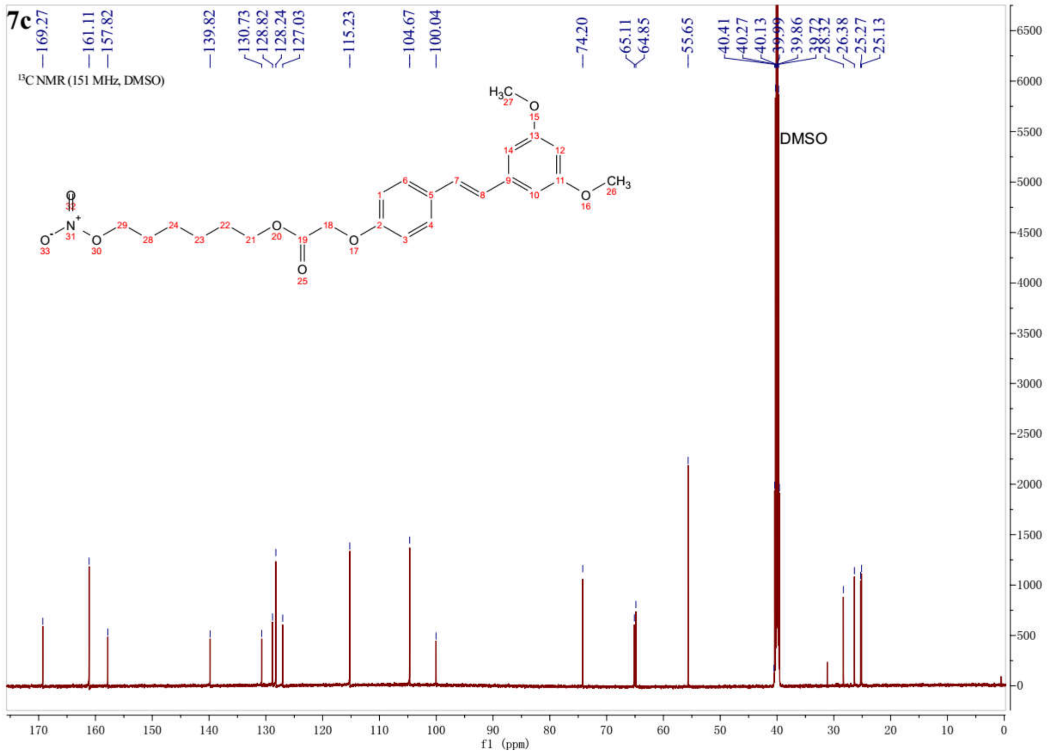
7c
^{13}C NMR (151 MHz, DMSO)
169.27
161.11
157.82
139.82
130.73
128.82
128.24
127.03
115.23
104.67
100.04
74.20
65.11
64.85
55.65
40.41
40.27
40.13
39.86
26.38
25.27
25.13
DMSO
f1 (ppm)
